# RNA editing by ADAR1 regulates innate and antiviral immune functions in primary macrophages

**DOI:** 10.1038/s41598-017-13580-0

**Published:** 2017-10-17

**Authors:** Maria Pujantell, Eva Riveira-Muñoz, Roger Badia, Marc Castellví, Edurne Garcia-Vidal, Guillem Sirera, Teresa Puig, Cristina Ramirez, Bonaventura Clotet, José A. Esté, Ester Ballana

**Affiliations:** 1 AIDS Research Institute-IrsiCaixa and Health Research Institute Germans Trias i Pujol (IGTP), Hospital Germans Trias i Pujol, Universitat Autònoma de Barcelona, Badalona, Spain; 2 HIV Clinical Unit, University Hospital Germans Trias i Pujol, Universitat Autònoma de Barcelona, Badalona, Spain

## Abstract

ADAR1-dependent A-to-I editing has recently been recognized as a key process for marking dsRNA as self, therefore, preventing innate immune activation and affecting the development and resolution of immune-mediated diseases and infections. Here, we have determined the role of ADAR1 as a regulator of innate immune activation and modifier of viral susceptibility in primary myeloid and lymphoid cells. We show that ADAR1 knockdown significantly enhanced interferon, cytokine and chemokine production in primary macrophages that function as antiviral paracrine factors, rendering them resistant to HIV-1 infection. ADAR1 knockdown induced deregulation of the RLRs-MAVS signaling pathway, by increasing MDA5, RIG-I, IRF7 and phospho-STAT1 expression, an effect that was partially rescued by pharmacological blockade of the pathway. In summary, our results demonstrate a role of ADAR1 in regulating innate immune function in primary macrophages, suggesting that macrophages may play an essential role in disease associated to ADAR1 dysfunction. We also show that viral inhibition is exclusively dependent on innate immune activation consequence of ADAR1 knockdown, pointing towards ADAR1 as a potential target to boost antiviral immune response.

## Introduction

Adenosine deaminases acting on RNA (ADAR) catalyze the conversion of adenosine (A) to inosine (I) in double-stranded RNA (dsRNA) substrates, a process of broad physiologic importance^1,2^. Three ADAR enzymes (ADAR1-3) are present in humans, albeit ADAR1 has been shown to play more significant roles in biological and pathological conditions, including infection, autoimmune disease and cancer^3^. Of note, recent data have identified ADAR1 function as a relevant factor in the regulation of the innate immune response [reviewed in^4^], suggesting that without appropriate RNA editing by ADAR1, endogenous RNA transcripts may stimulate cytosolic RNA sensing receptors and therefore activate the IFN-inducing signaling pathways^5^. Growing evidences support a role for A-to-I editing in dsRNA by ADAR1 in the replication process of different viruses. However, ADAR1 function has been associated either to enhanced or reduced virus growth or persistence, depending upon the specific virus-host combination^6^. ADAR1 has emerged as a replication enhancer of retroviruses, including the human immunodeficiency virus (HIV-1) during acute infections^6^. The proviral role of ADAR1 has been associated to two distinct mechanisms; (i) editing of viral substrates^7–9^ or (ii) an editing-independent process that may be linked to the inhibition of the double-stranded RNA-dependent protein kinase (PKR)^10–12^. However, if the two mechanisms coexist and/or cooperate to increase viral replication in innate immune cells is unclear.

The innate immune system provides the first line of defense against infection, primarily by intracellular recognition of foreign nucleic acids, leading to increased production of proinflammatory cytokines, chemokines, and interferons (IFN)^13,14^. Deficiencies in these mechanisms can predispose to viral infections and precipitate autoimmune diseases. Much of our understanding of the pathways associated to aberrant IFN production comes from the genetic dissection of the Aicardi-Goutières syndrome (AGS), a severe human autoimmune disease. AGS is characterized by a phenotype resembling a congenital infection, including high levels of IFN in cerebrospinal fluid and serum as one of the critical hallmarks of the disease^15,16^. Mutations in seven human genes have been identified in AGS patients: *TREX1*, *RNASEH2A*, *RNASEH2B*, *RNASEH2C*, *SAMHD1*, *ADAR1* and *IFIH1*, all being nucleic acid-catabolizing proteins that participate in pathways for sensing and/or clearing away foreign and self-derived nucleic acids^17^.

The innate immune system is dependent on various cell types such as dendritic cells (DCs) and macrophages that are capable of detecting invading pathogens, responding to infections through secretion of proinflammatory cytokines, chemokines, and IFNs, upregulating expression of co-stimulatory molecules, and processing and presenting antigens to naïve T cells to activate the adaptive immune system^13^. The rapid production of IFN is a cornerstone of the innate antiviral response. IFN expression is dependent upon cellular transcription factors, including interferon regulatory factor (IRF)-3 and IRF-7, which become activated as downstream effectors of pathogen recognition receptor (PRR) signaling cascades. PRR include the RNA-binding helicase family of RIG-I-like receptors (RLR: RIG-I and MDA5), and the family of Toll-like receptors (TLR). IRF3 is constitutively expressed in most cells and serves to induce an initial wave of IFN expression, amplified upon subsequent expression and activation of IRF7, which itself is an interferon stimulated gene (ISG)^18^.

Here, we determined the role of RNA editing of ADAR1 in distinct immune cells as a regulator of innate immune activation and modifier of viral susceptibility. Overall our results demonstrate an important role of A-to-I editing in dsRNA by ADAR1 in regulating innate immune function in primary macrophages, provide novel insights for a better comprehension of the innate immune mechanisms that affect intracellular recognition of nucleic acids and point to ADAR1 as a potential target to boost antiviral immune response.

## Results

### ADAR1 function regulates type I IFN and innate immune activation in primary macrophages

**Figure 1:**
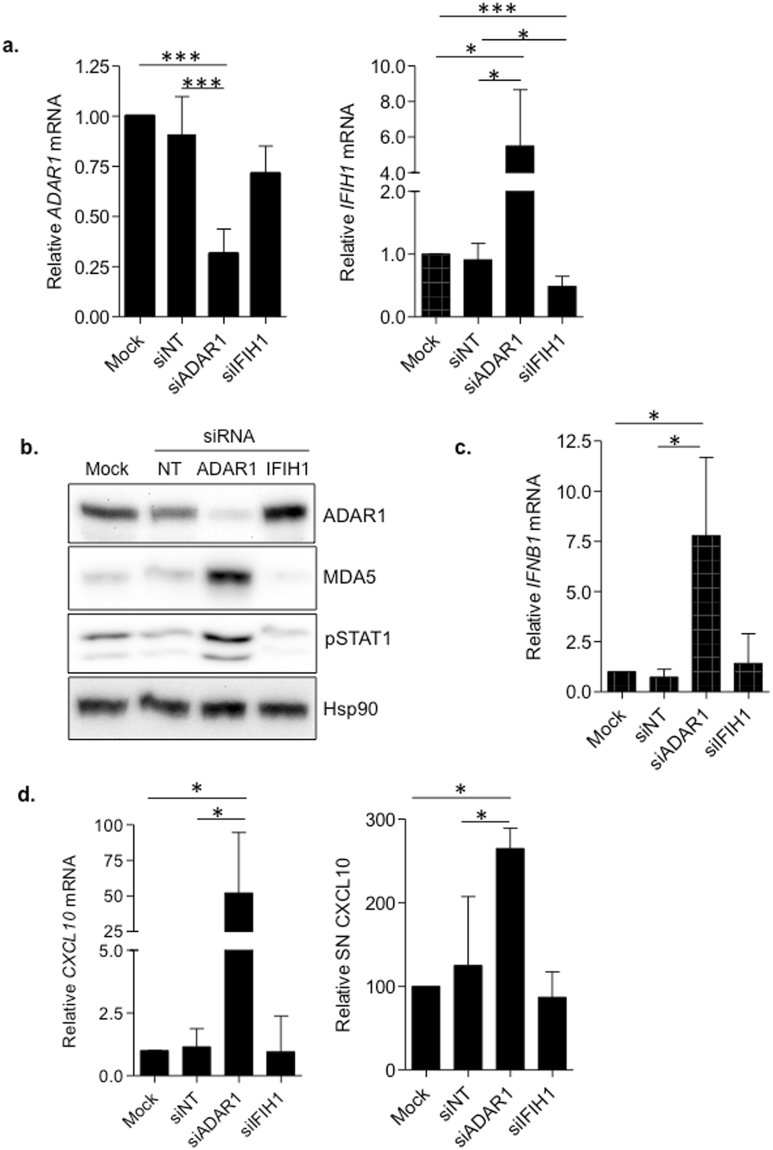
ADAR1 knockdown boosts type I IFN response in primary macrophages. **(a)** Gene expression of *ADAR1* (left panel) and *IFIH1* (right panel) knockdown macrophages. Relative mRNA expression of *ADAR1* and *IFIH1* was measured by quantitative PCR and normalized to *GAPDH* expression. Data represents mean±SD of 5 different donors and is normalized to mock-transfected M-CSF macrophages. **(b)** Protein expression in ADAR1 and IFIH1 knockdown macrophages. Western blot of ADAR1, MDA5 and phosphorylation of STAT1 (pSTAT1) in siRNA-treated M-CSF macrophages. MDA5 and pSTAT1 are increased in *ADAR1* knockdown macrophages compared to the corresponding non-targeting siRNA (NT). Hsp90 was used as loading control. A representative donor is shown. The figure shows the cropped gels/blots obtained by each protein evaluation. Full-length blots of each tested protein are included in supplementary material. **(c)** Relative mRNA expression of IFNβ in siRNA-treated macrophages measured by quantitative PCR and normalized to GAPDH expression. *IFNB1* gene expression was significantly enhanced in *ADAR1* knockdown macrophages. Data represents mean ± SD of 5 different donors and is normalized to mock-transfected M-CSF macrophages. **(d)** CXCL10 mRNA (left panel) and protein expression in the supernatant (right panel) in siRNA-treated macrophages. Relative mRNA expression of *CXCL10* was measured by quantitative PCR and normalized to GAPDH expression. CXCL10 protein in the culture supernatants was measured by ELISA. Data represents mean ± SD of 3 different donors. In all panels, isolated monocytes were transfected with the corresponding siRNA and differentiated to macrophages with M-CSF for 4 days. *p < 0.05; **p < 0.005; ***p < 0.0005.

To investigate the regulation of the innate immune response we used *in vitro* differentiated primary macrophages^19–25^. Once isolated from donor PBMCs, monocytes were transfected with with small interfering RNA (siRNA) targeting *ADAR1* and *IFIH1* (MDA5) and further differentiated to macrophages with M-CSF^19,20,22–25^. Effective and specific downregulation of ADAR1 and *IFIH1* (MDA5) was achieved at both mRNA and protein level in macrophages (Fig. 1a and b). ADAR1 downregulation led to a significantly enhanced expression of the cytosolic RNA sensor MDA5, both at mRNA (5.5-fold change, p = 0.0001) and protein levels (Fig. 1a and b), as previously suggested^5^. ADAR1 knockdown macrophages also showed increased production of IFNβ (7.5-fold change, p = 0.0388, Fig. 1c) and the IFN stimulated gene (ISG) CXCL10, measured by mRNA expression (50-fold change, p = 0.049, Fig. 1d) and as a secreted chemokine in the culture supernatant (2.6-fold change, p = 0.01, Fig. 1d). In addition, increased phosphorylation of STAT1 (pSTAT1), another well-recognized marker of type I IFN induction, was observed in ADAR1 knockdown macrophages (Fig. 1b). In contrast, no effect was seen in MDA5 knockdown macrophages (Fig. 1a–d). Importantly, confirmatory siRNA sequences targeting ADAR1 showed similar effects in activation of type I IFN response (Supplementary Fig. S1A and B). These data are suggestive of a role of ADAR1 as a negative regulator of innate immune response in primary macrophages.

### ADAR1 downregulation blocks HIV-1 transcription in primary macrophages

**Figure 2:**
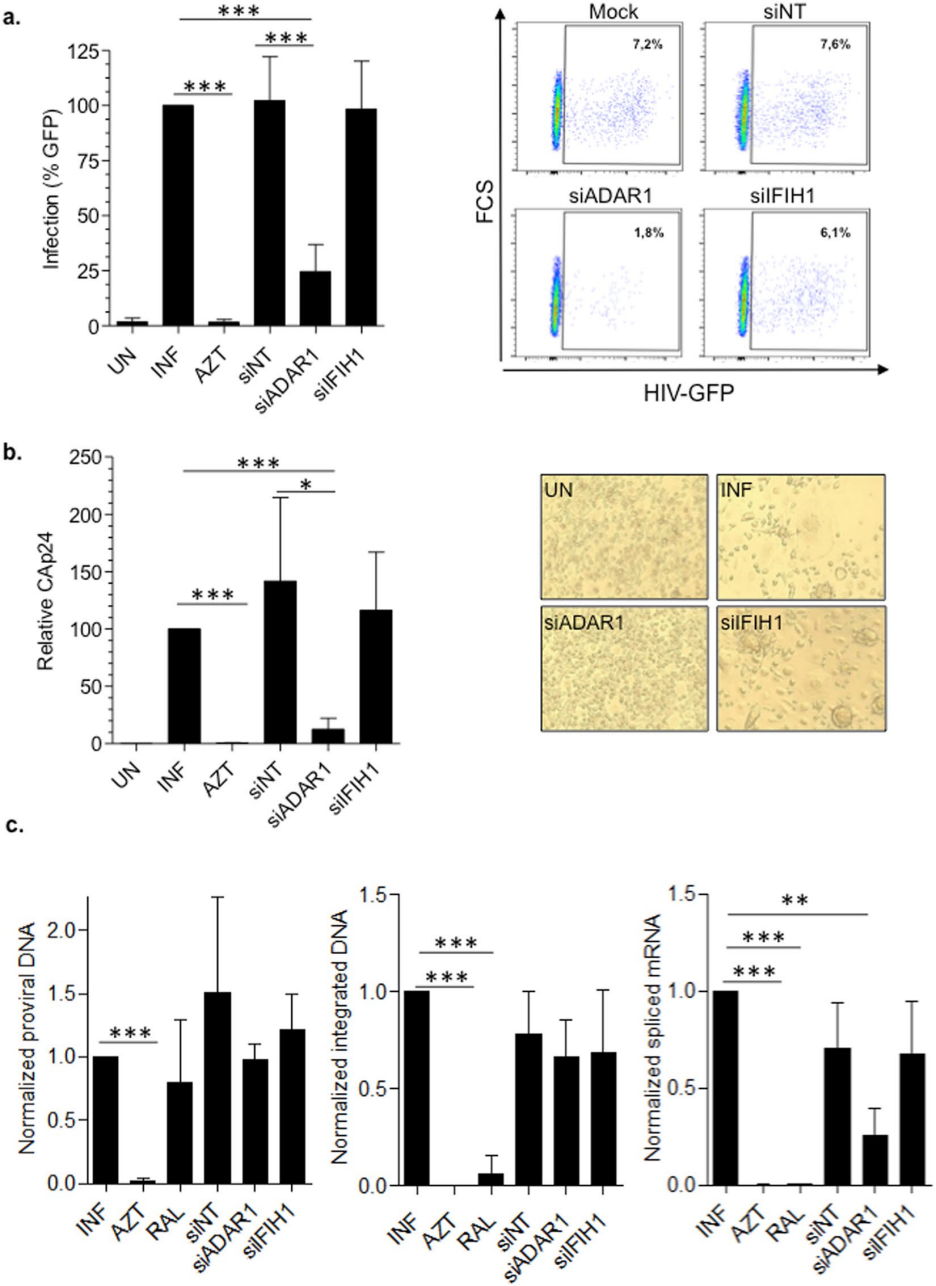
ADAR1 knockdown blocks HIV-1 transcription in primary macrophages. (**a**) HIV-1 replication in *ADAR1* and *IFIH1* knockdown M-CSF macrophages, infected with a VSV-pseudotyped, GFP-expressing HIV-1. Infection was measured 72 h later by flow cytometry. Data represent percentage replication relative to mock-transfected macrophages (left panel). A representative flow cytometry dot plot showing infected macrophages is also depicted (right panel). Mean ± SD of at least 5 different donors performed in triplicate is shown. **(b)** HIV-1 replication in ADAR1 and IFIH1 knockdown M-CSF macrophages, infected with a full replicative HIV-1 BaL strain. Differentiated macrophages were infected for 7 days and CAp24 production was measured in culture supernatant by ELISA. Mean ± SD of 3 different donors performed in triplicate is shown. **(c)** Proviral DNA formation (left panel), viral integration (middle panel) and viral transcription (right panel) in ADAR1 and IFIH1 knockdown macrophages. siRNA-treated and subsequently differentiated macrophages were infected with HIV-1 BaL for 16 h (for proviral DNA formation), 20 h (for viral DNA integration) or 40 h (for viral transcription) before DNA or RNA extraction. All determinations were normalized to mock-treated infected macrophages and AZT (3 µM) or raltegravir (RAL; 2 µM), were included as controls. Mean ± SD of at least 3 different donors is shown. In all panels, isolated monocytes were transfected with the corresponding siRNA and differentiated to macrophages with M-CSF for 4 days, prior to infection with HIV-1- *p < 0.05; **p < 0.005; ***p < 0.0005.

The role of ADAR1 on HIV-1 susceptibility of primary macrophages was evaluated by testing the capacity of siRNA-treated macrophages to support HIV-1 replication. Significant inhibition of HIV-1 replication was seen in *ADAR1* knockdown macrophages either using a VSV-pseudotyped single cycle GFP-expressing HIV-1 (roughly 75% inhibition, p = 0.0001, Fig. 2a) or a full replicative R5 HIV-1 BaL (roughly 85% inhibition, p = 0.0001, Fig. 2b). Confirmatory siRNA sequences targeting ADAR1 showed comparable inhibition of HIV-1 replication (Supplementary Fig. S1C). Conversely, inhibition of *IFIH1* (MDA5) expression did not have any effect on HIV-1 infection (Fig. 2a and b).

Thus, we investigated the specific step of viral replication cycle affected by ADAR1 knockdown. For this purpose, the levels of total viral DNA, integrated proviral DNA and multispliced viral transcripts were quantified by qPCR. No significant changes were observed in proviral DNA formation or integration in *ADAR1* or *IFIH1* knockdown macrophages (Fig. 2c, left and middle panels). As expected, the HIV-1 reverse transcriptase inhibitor AZT completely blocked viral DNA formation, while the HIV-1 integrase inhibitor raltegravir (RAL) did not have any effect on viral DNA formation, but inhibited viral integration (Fig. 2c, left and middle panels). Conversely, HIV-1 transcription was significantly inhibited in *ADAR1* knockdown macrophages (p = 0.0007, Fig. 2c, right panel), indicating that ADAR1 function is necessary for appropriate HIV-1 transcription.

ADAR1 may be affecting viral replication by directly A-to-I editing of HIV-1 mRNAs^7^. However, no A-to-I editing was detected in five different HIV-1 mRNA predicted editing sites of infected macrophages, while editing of cellular mRNAs was clearly seen in siNT macrophages but not in ADAR1 knockdown macrophages (Supplementary Fig. S2), demonstrating that ADAR1 is functional in primary macrophages. Besides its catalytic function, ADAR1 can also serve an editing-independent role that relies on the inhibition of PKR, an IFN-induced protein reported to inhibit mRNA translation^11^. As expected, PKR expression was enhanced in ADAR1 knockdown macrophages (Supplementary Fig. S3A), validating previous data^11^. However, PKR knockdown did not have any effect on HIV-1 replication or innate immune function (Supplementary Fig. S3B–D). ADAR1 knockdown macrophages infected with the full replicative HIV-1 Bal strain for 7 days maintained the enhanced expression of IFNβ and CXCL10 (Supplementary Fig. S4).

Altogether, these results demonstrate that direct A-to-I posttranscriptional editing of viral mRNA or PKR induction may not be the underlying mechanisms of ADAR1 effect on HIV replication; pointing towards the regulation of innate immune function as the key process affecting viral replication.

### ADAR1-mediated innate immune activation and block of HIV-1 infection is specific of macrophages

**Figure 3:**
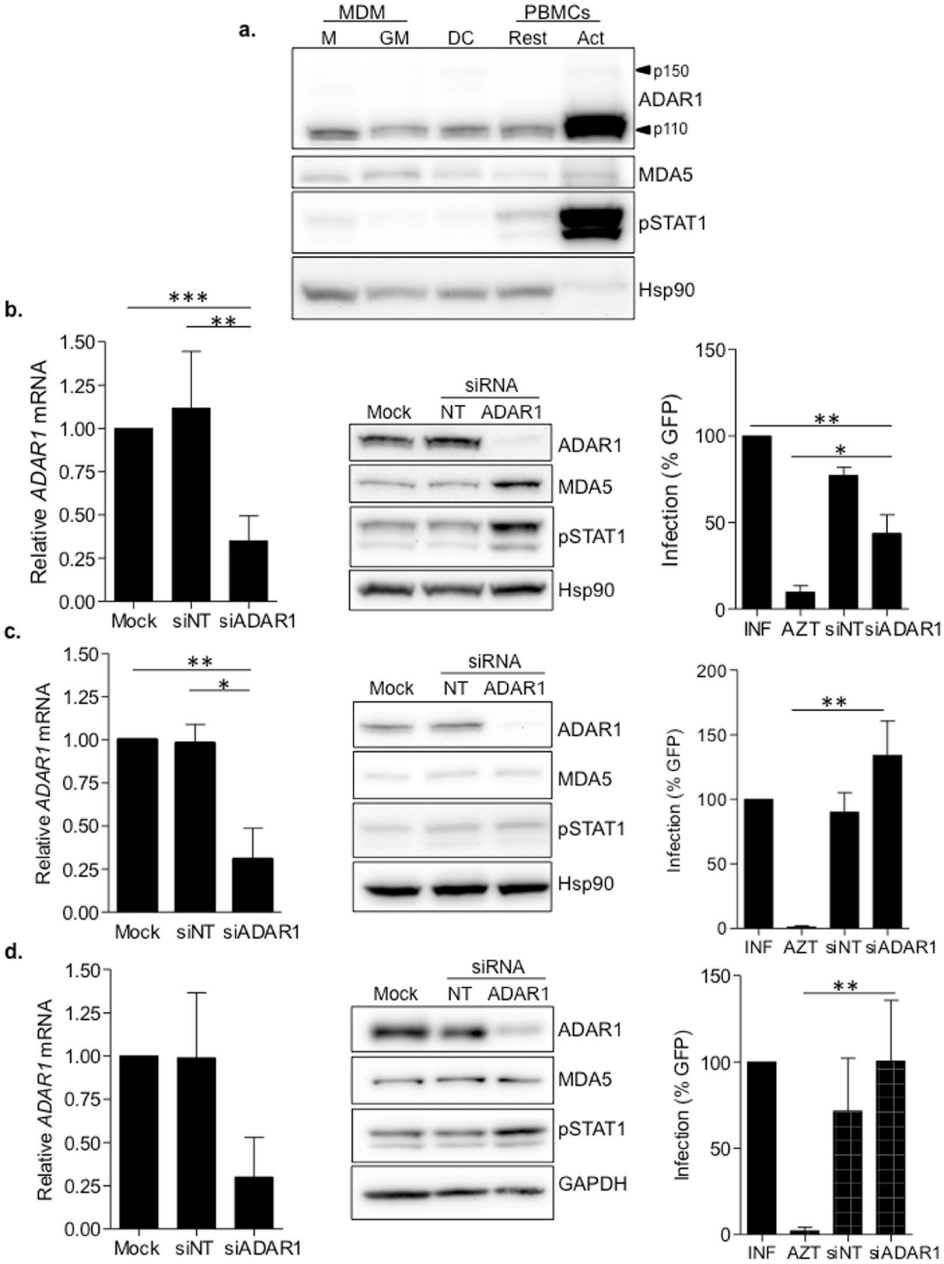
ADAR1-mediated regulation of innate immune activation and HIV-1 infection is specific of macrophages. (**a**) Evaluation of ADAR, MDA5 and pSTAT1 protein expression in different myeloid and lymphoid primary cells. ADAR1 and pSTAT1 expression were increased in activated PBMCs compared to other cell types. M; M-CSF macrophages, GM; GM-CSF macrophages, DC; monocyte derived dendritic cells, Rest; Resting PBMCs and Act, Activated PBMCs. Hsp90 was used as loading control. A representative experiment is shown. (**b–d**) Evaluation of ADAR1 knockdown in GM-CSF macrophages (**b**), monocyte-derived dendritic cells (**c**) and CD4+ T cells (**d**). Downregulation of ADAR1 mRNA expression by qPCR (left panels), western blot showing protein expression (middle panels) and susceptibility to HIV-1 infection (right panels) are shown. Effective ADAR1 mRNA inhibition was achieved in all cell types (B-D, left panels). Upregulation of MDA5 and pSTAT1 was only observed in ADAR1 knockdown GM-CSF macrophages (**b**, middle panel), which correlated with inhibition of HIV-1 replication (B, right panel). No significant differences were observed in protein expression or HIV-1 infection in dendritic cells or CD4+ T cells, following ADAR1 knockdown (C and D, middle and right panels respectively). Data from mRNA expression and HIV-1 infection represent the mean ± SD of 3 different donors. A representative western blot is shown in each case. The figure shows the cropped gels/blots obtained by each protein evaluation. Full-length blots of each tested protein are included in supplementary material. *p < 0.05; **p < 0.005.

M-CSF macrophages represent an *in vitro* model of an HIV-1 target cell, relevant for innate immune function. However, the innate immune system is dependent on other cell types. Thus, we aimed at investigating the role of ADAR1 in other primary cells from the myeloid compartment and CD4+ T-cells. Protein expression of ADAR1, MDA5 and phosphorylation of STAT1 (pSTAT1) were evaluated in M-CSF and GM-CSF monocyte-derived-macrophages, monocyte derived dendritic cells (moDC) and resting and activated PBMCs. All cell types showed comparable expression of ADAR1, MDA5 and pSTAT1, except for the higher expression of ADAR1 and pSTAT1, observed in activated PBMCs (Fig. 3a). Then, ADAR1 expression was downregulated using siRNA and the effect on type I IFN expression and susceptibility to HIV-1 infection was evaluated. Effective and specific downregulation of ADAR1 was achieved in all cell types tested (GM-CSF macrophages, moDC and CD4+ T-cells, Fig. 3b–d, left panels). However, induction of MDA5 expression or phosphorylation of STAT1 after ADAR1 knockdown was only observed in GM-CSF macrophages, and slightly in CD4+ T cells, but not in other cell types (Fig. 3b–d, middle panels). Evaluation of *IFNB1* and *CXCL10* mRNA expression also confirmed that inhibition of ADAR1 affected the innate immune function on GM-CSF macrophages, but not in moDC (Supplementary Fig. S5). Indeed, HIV-1 infection was only significantly inhibited in GM-CSF macrophages (roughly 60% inhibition, p = 0.0019, Fig. 3b, right panel), whereas no change in HIV-1 susceptibility was seen in moDC or CD4+ T lymphocytes (Fig. 3c and d, right panels). Therefore, ADAR1-mediated innate immune regulation appears to be especially relevant in macrophages, but not in other cell types.

### ADAR1 is a negative regulator of the RIG-I like receptor (RLRs)-MAVS signaling pathway

As demonstrated above, ADAR1 is a cofactor of HIV-1 replication in primary macrophages which presumably acts as a negative regulator of innate immune response. Therefore, we aimed to further explore the intracellular signaling pathway that leads to innate immune activation following ADAR1 knockdown.

**Figure 4:**
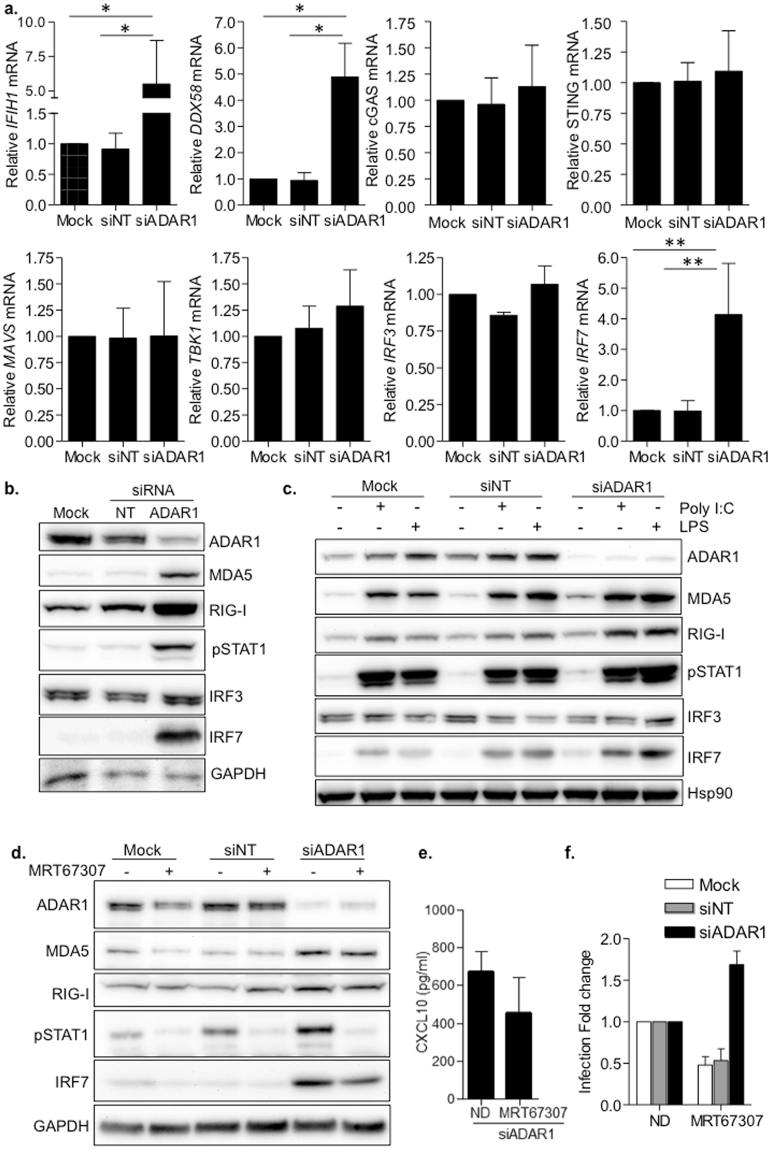
ADAR1 specifically regulates the RLRs-MAVS signaling pathway. (**a**) Relative mRNA expression of RLRs (*IFIH1* and *DDX58*), DNA sensors (cGAS and STING), downstream signaling proteins (*MAVS*, *TBK1*) and transcription factors (*IRF3* and *IRF7*) in ADAR1 knockdown macrophages. Data represents mean ± SD of at least 4 different donors and is normalized to Mock-transfected macrophages. *p < 0.05; **p < 0.005. **(b)** Protein expression of RLRs and related proteins in macrophages, showing overexpression of MDA5, RIG-I, pSTAT1 and IRF7 consequence of ADAR1 inhibition. A representative donor is shown. **(c)** Western blot showing protein expression pattern in transfected macrophages, stimulated or not with LPS (100 ng/ml) or Poly I:C (10 µg/ml) to resemble canonical TLR-mediated activation of type I IFN response. A representative donor is shown. **(d)** Protein expression in ADAR knockdown macrophages treated with the TBK1 inhibitor MRT67307 (5 µM). Blocking TBK1 function partially restores protein expression phenotype observed in Mock- or siNT-transfected macrophages. A representative donor is shown. **(e)** CXCL10 protein expression in the supernatant in ADAR1 knockdown macrophages, treated or not with MRT67307 (5 µM). CXCL10 protein in the culture supernatants was measured by ELISA. Data represents mean ± SD of 3 different donors. **(f)** Change in HIV-1 replication of macrophages treated with the TBK1 inhibitor MRT67307 (5 µM). Fold change of HIV-1 infection in macrophages treated or not with MRT67307. Infection is normalized to the corresponding untreated condition. Data represents mean ± SD of 3 different donors performed in triplicate. *p < 0.05; **p < 0.005. The figure shows the cropped gels/blots obtained by each protein evaluation. Full-length blots of each tested protein are included in supplementary material.

ADAR1 knockdown led to a significant increase in the RNA sensors *IFIH1* (MDA5) and *DDX58* (RIG-I) and the interferon regulatory factor *IRF7*, compared to mock transfected macrophages or macrophages treated with a non-targeting siRNA (siNT) (p = 0.0129 and p = 0.0024 respectively, Fig. 4a). Conversely, no changes in mRNA expression of DNA cytosolic sensors cGAS and STING or the downstream effectors *MAVS*, *TBK1* and *IRF3* were observed. There was a clear correlation between ADAR1 knockdown and overexpression of MDA5, RIG-I, IRF7 protein expression and the phosphorylation of STAT1 (Fig. 4b, Supplementary Fig. S1D, Supplementary Fig. S6A). Stimulation of macrophages with LPS or poly I:C showed a similar pattern of protein overexpression (Fig. 4c, Supplementary Fig. 6A), suggesting that ADAR1 is a regulator of the RLRs-MAVS canonical pathway of innate immune activation leading to type I IFN production.

MRT67307, an inhibitor of IKKϵ and TBK1, has been shown to prevent the phosphorylation of IRF3 and the production of IFNβ in macrophages^26^. Treatment of ADAR1 knockdown macrophages with MRT67307 limited the effects of ADAR1 depletion by significantly reducing overexpression of IRF7 and pSTAT1 and to a lesser extent RLR (Fig. 4d), and its effect was dose-dependent (Supplementary Fig. S6B). As expected, a decrease in the amount of secreted CXCL10 in siADAR1 macrophages in the presence of MRT67307 was also observed (4E), indicating that MRT67307 limits innate immune activation associated to ADAR1 downregulation. Moreover, although MRT67307 was able to partially inhibit HIV-1 infection in mock or siNT macrophages, this was not observed in ADAR1 knockdown cells (Fig. 4f). These data suggest that ADAR1 function specifically regulates de RLRs-MAVS signaling pathway and that innate immune activation consequence of ADAR1 inhibition is the responsible of the block in HIV-1 replication.

### Immune activation in siADAR1 macrophages is pro-inflammatory and inhibits HIV replication in bystander macrophages

Once transcriptional activation of type I IFN genes has taken place, IFN and cytokines are produced and secreted. Secreted IFNs may act as autocrine and paracrine factors and initiate a tissue-wide, systemic signaling through the cellular Jak-STAT pathway, inducing the transcription of hundreds of ISG^27^. Therefore, the type of cytokines produced by ADAR1 knockdown macrophages was further characterized and their paracrine function in terms of antiviral activity was evaluated.

**Figure 5:**
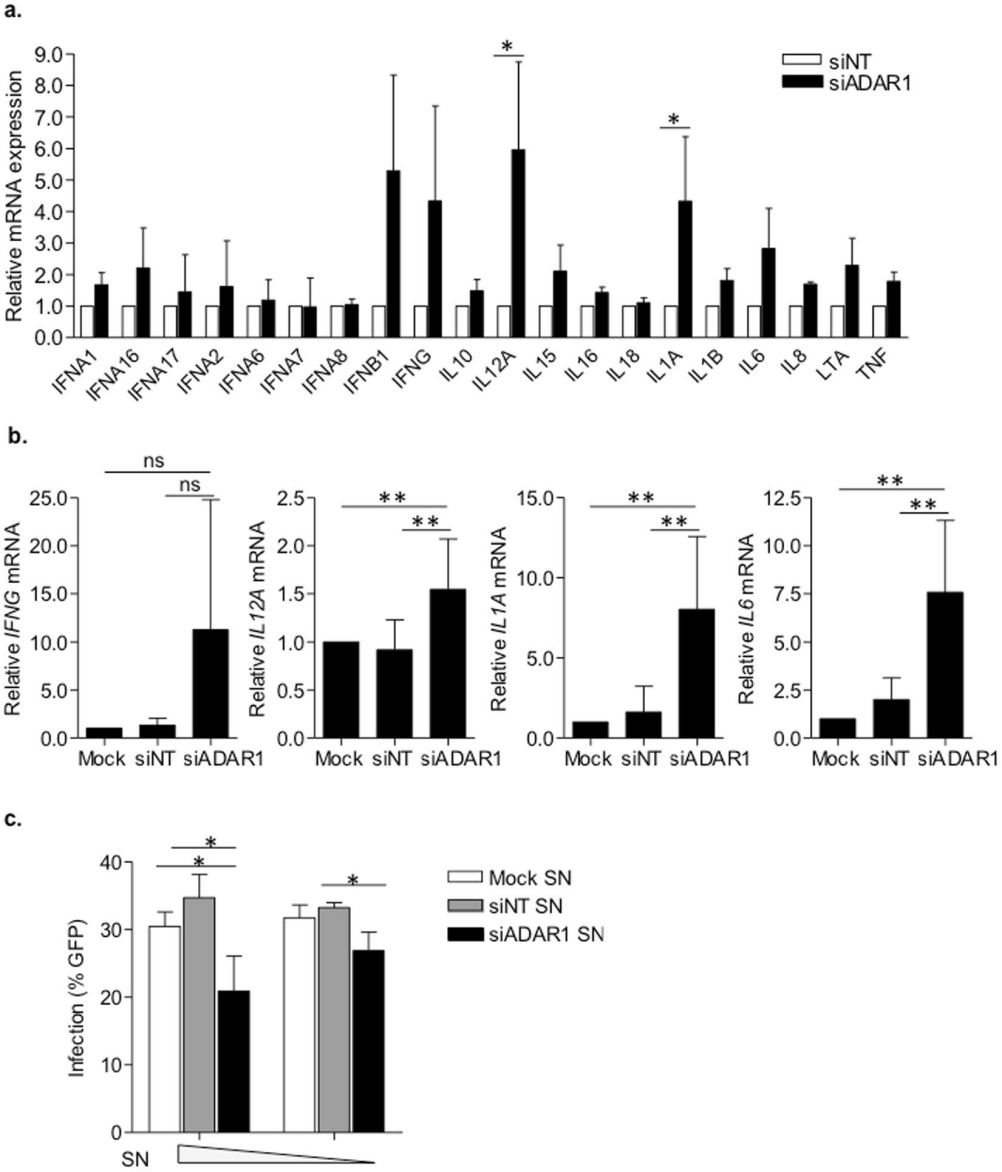
ADAR1 knockdown macrophages present a pro-inflammatory phenotype that inhibits HIV-1 replication of bystander cells. (**a**) Cytokine expression profile of ADAR1 knockdown macorphages. Relative mRNA expression of the different cytokines normalized to siNT-transfected macrophages is depicted. Data represents mean ± SD of 3 different donors. *p < 0.05. **(b)** Relative mRNA expression of cytokines identified as upregulated in ADAR1 knockdown macrophages *(IFNG*, *IL12A*, *IL1A* and *IL6*). Data represents mean ± SD of 6 independent donors and is normalized to mock-transfected macrophages. ns; non-significant, **p < 0.005. **(c)** Antiviral activity of culture supernatants from ADAR1 knockdown macrophages. Percentage infection of untreated macrophages incubated with different concentrations of culture supernatants from mock- (white bars), siNT- (grey bars) and siADAR1- (black bars) treated macrophages. SN; supernatant, *p < 0.05.

We evaluated the contribution of IFNβ to ADAR1-mediated inhibition of HIV-1 replication in the presence of a blocking antibody targeting IFNβ. However, no change in HIV-1 susceptibility was observed in ADAR1 knockdown macrophages, in mock-transfected macrophages or macrophages transfected with a non-targeting siRNA (Supplementary Fig. S7). However, apart from *IFNB1*, four other genes (*IFNG*, *IL12A*, *IL1A* and *IL6*) showed enhanced expression in ADAR1 knockdown macrophages (at least 2.5 fold difference compared to siNT-treated macrophages, Fig. 5a), an effect that was further confirmed in six additional donors (Fig. 5b). Upregulated cytokines are suggestive of a pro-inflammatory macrophage phenotype with an inhibitory effect on HIV-1 replication. Thus, antiviral activity of culture supernatants from transfected macrophages was evaluated in primary macrophages. Culture supernatants from ADAR1 knockdown macrophages inhibited HIV-1 replication in a dose-dependent manner (roughly 35% inhibition at highest concentration, p = 0.05, Fig. 5c), whereas no effect was seen in mock or siNT transfected macrophages. Although the inhibitory capacity of supernatants from ADAR1 knockdown macrophages is modest compared to ADAR1 depletion, these results indicate that pro-inflammatory cytokines from ADAR1 knockdown macrophages are secreted and can function as paracrine factors that contribute to HIV-1 susceptibility.

## Discussion

To distinguish self from non-self is a core mission of the immune system^28^. ADAR1-dependent A-to-I editing has recently been recognized as a key process for marking dsRNA as self, therefore, preventing innate immune recognition by cytosolic sensors and, in consequence, autoreactivity and the development of inflammation and/or autoimmune disease^4^. Here, we describe the role of ADAR1 in regulating innate immune function in primary macrophages and how its deregulation changes susceptibility to HIV-1 infection.

ADAR1 has been proposed to be a critical suppressor of IFN responses, which protects cells from the harmful effects of excessive IFN signaling^4^. In line with these observations, we found that inhibition of ADAR1 expression in primary macrophages significantly enhance type I IFN production. Indeed, the presence of high levels of IFN in cerebrospinal fluid and serum from AGS patients has been identified as one of the critical hallmarks of the disease^15^. Moreover, a high percentage of AGS patients present also increased level of ISGs in peripheral blood^16,29,30^. Similar results were obtained from transcriptome analysis of Adar1 knockout mice^31,32^ or mice with an editing deficient knock-in mutation Adar1^E861A^ ^5^, both presenting embryonic lethality in homozygotes due to gene expression signature of type I and type II ISGs. Consistent with this, our study shows that silencing ADAR1 in macrophages results in an increased expression of proinflammatory cytokines, indicating that our macrophage *in vitro* model resembles innate immune activation features observed in AGS patients and mice models *in vivo*. Additionally, it also demonstrates in a human primary cell culture that ADAR1 function is mediated by the concurrent effects of numerous edited substrates and tightly linked to innate immune response.

The exact mechanism by which ADAR1 functions is still far from understood, despite intensive research particularly in ADAR1 genetically modified mice^5,31,32^. Liddicoat *et al*. linked RNA editing to RNA recognition exclusively by cytosolic sensor MDA5, based on the fact that MDA5 deficiency could rescue the phenotype of Adar1^E861A/E861A^ editing-deficient mice^5^. However, two major sensors of cytosolic dsRNA exist, MDA5 and RIG-I, with different RNA chain preferences^33–35^. The type of dsRNA described by Liddicoat *et al*. does not fit into the knowledge of dsRNA preference by RIG-I and MDA5. Moreover, an Adar1 knockout model indicated that Mavs deficiency, an essential adaptor of both RIG-I and MDA5, was not able to totally revert the Adar1 knockout phenotype^36^, by turning off the signaling cascade. These results suggest that apart from the MDA5-MAVS axis, additional mechanisms mediate innate immune response in the absence of ADAR1. In our cell culture, both cytosolic dsRNA receptors MDA5 and RIG-I are similarly affected by ADAR1 knockdown, comparable to LPS or poly (I:C) induction, indicating that the effect might not be exclusively driven by MDA5. Moreover, pharmacological blockade of TBK1^26^ partially rescued the phenotype observed in ADAR1 knockdown macrophages, indicating that ADAR1 deficiency may signal through MAVS-TBK1.

**Figure 6:**
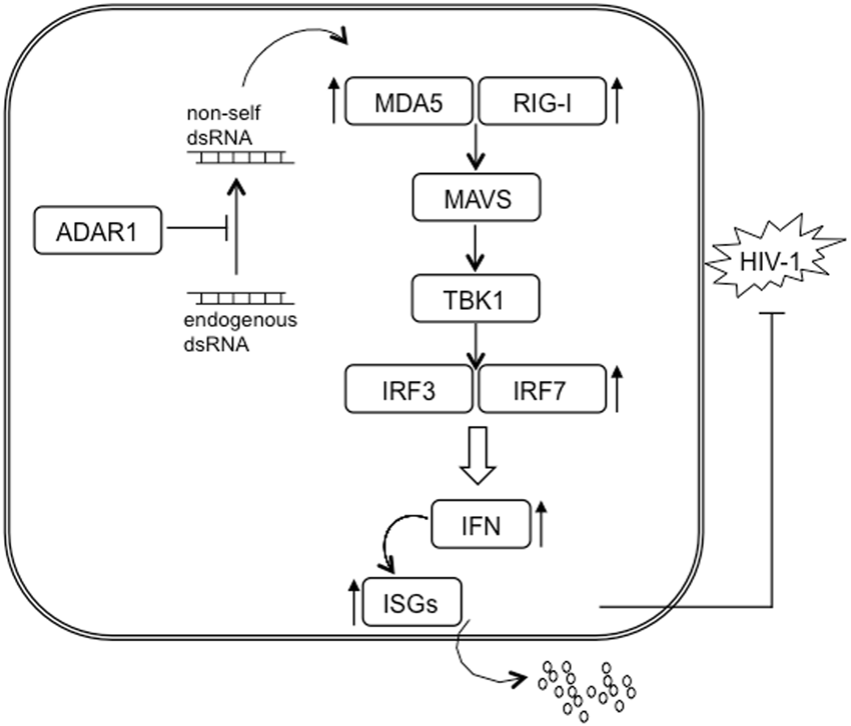
Representation of signaling cascade regulated by ADAR1 in MDM. ADAR1 modulates the recognition of non-self dsRNA in the cytoplasm; consequently, sensing of non-self dsRNA induces expression of RNA sensors, RIG-I and MDA5, through downstream effectors, MAVS and TBK1, and transcription factors IRF3 and IRF7. Leading to induction of an innate immune response characterized by increase expression of type I IFN and ISG and subsequent over production of cytokines and chemokines able to block HIV-1.

Apart from endogenous sources of dsRNA, foreign or viral dsRNA may also trigger immune responses via the RLR-MAVS pathway. Thus, it is not surprising that viral cofactors, such as ADAR1 are tightly connected with innate immune recognition of nucleic acids and the subsequent induction of type I IFN. As suggested by others, our results also demonstrate the proviral role of ADAR1 in HIV-1 infection, *i.e*., downregulation or knockdown of ADAR1 is deleterious for viral replication^7,10,37^. However, the underlying mechanism is still controversial, and conflicting mechanisms has been suggested, ranging from direct editing of HIV-1 mRNA, towards editing-independent effect mediated by the IFN-inducible protein PKR affecting viral translation^6^. We did not detect A-to-I editing in viral mRNAs, previously suspected as targets for ADAR1 function^7^. However, we cannot exclude the existence of edited sites in other viral genomic regions or a cell dependent-effect, as reported elsewhere^7^. PKR inhibits mRNA translation and ADAR1-mediated PKR inhibition has been reported during measles virus, vesicular stomatitis virus, HTLV-1 infection and HIV-1^11,38–41^. In contrast to previous data, our results do not support a direct role of PKR in HIV-1 replication in macrophages as PKR inhibition is not affecting HIV-1 replication and ADAR1 knockdown blocks HIV-1 at viral transcription, prior to viral translation. Therefore, we suggest that the concerted ADAR1-PKR regulation might be exclusively dependent on the induction of IFN production consequence of ADAR1 dysfunction. Taken into account the notion that ADAR1 function is mediated by the concurrent effects of numerous edited substrates, including cellular but also viral RNAs, our data indicate that RNA editing by ADAR1 prevents innate immune activation and the subsequent IFN production and this is the underlying mechanism explaining ADAR1 proviral role in HIV-1 infection (Fig. 6). Thus, ADAR1 may be controlling the activation of an antiviral state, which may imply the modification of certain viral mRNAs, and can maintain PKR expression at basal levels. Indeed, HIV-1 infection itself is able to induce RLR-MAVS signaling pathway in long-term macrophage infection, therefore providing further evidences of the important role of innate immunity in macrophages^23^.

Interestingly, the innate immune activation marks and subsequent inhibition of HIV-1 replication observed in macrophages (both M-CSF and GM-CSF derived) were not observed when ADAR1 was downregulated in DC or only partially observed in CD4+ T cells. This data is in contrast with previous data from ADAR1-deficient CD4+ T lymphocytes from AGS patients^37^, which presented high type I IFN and ISG expression and were also refractory to HIV-1 infection. The lack of innate immune activation in our CD4+ T cell cultures compared to that observed in CD4+ T cells from AGS patients may account for the observed differences^37^, reinforcing the idea that ADAR1-mediated block of HIV-1 replication is mainly dependent on innate immune activation. On the other hand, distinct expression patterns of innate immune receptors in different cell types and cell-type-dependent differences in the expression of downstream signaling components and transcription factors contribute to the complexity of nucleic acid immunity^17^, implying different types of response from a single stimulus. Furthermore, the effect of AGS gene deficiency seems to be both tissue and cell specific^42^, putatively explaining the differences observed after ADAR1 downregulation between cell types. Importantly, our work indicate that macrophages might have a relevant role in the pathogenesis associated to AGS patients with ADAR1 mutations, at least in comparison to other immune cell types and also provides an *in vitro* model in primary cells that may be useful for further investigations on innate immune processes.

In summary, we here describe how the lack of RNA editing by ADAR1 results in the activation of innate immune response leading to the inhibition of viral infection. Our results provide novel insights for a better comprehension of the innate immune mechanisms that affect intracellular recognition of nucleic acids and point to ADAR1 as a potential target to boost antiviral immune response in specific cell types. Furthermore, a better understanding of the molecular pathways of nucleic acid immunity and the functional interaction between them is expected to advance medicine specifically in the areas of infection and inflammation and with broad implication for human diseases.

## Methods

### Cells

Peripheral blood mononuclear cells (PBMC) were obtained from blood of healthy donors using a Ficoll-Paque density gradient centrifugation and CD4+ T lymphocytes or monocytes were purified using negative selection antibody cocktails (StemCell Technologies) as described before^19^. Monocytes were cultured in complete culture medium (RPMI 1640 medium supplemented with 10% heat-inactivated fetal bovine serum (FBS; Gibco) and penicillin/streptomycin (Gibco) and differentiated to monocyte derived macrophages (MDM) for 4 days in the presence of monocyte-colony stimulating factor (M-CSF, Peprotech) or granulocyte-macrophage colony-stimulating factor (GM-CSF, Peprotech) both at 100 ng/ml, or alternatively to monocyte derived dendritic cells (moDC) for 5 day in the presence of GM-CSF at 20ng/ml and IL-4 at 2ng/ml. CD4+ T cells were activated with anti-CD3 and anti-CD28 (at 1 µg/ml each, StemCell technologies) for 3 days.

The protocol was approved by the scientific committee of *Institut de Recerca de la Sida* - IrsiCaixa. Buffy coats were purchased from the *Catalan Banc de Sang i Teixits* (http://www.bancsang.net/en/index.html). The buffy coats received were totally anonymous and untraceable and the only information given was whether or not they have been tested for disease. All donors provided informed consent at the time of blood extraction. All methods were carried out in accordance with relevant guidelines and regulations and to the ethical principles suggested in the Declaration of Helsinki.

### RNA interference

Isolated monocytes were transfected as previously described^19,22,25^. Briefly, 50 pmol of the corresponding siRNA (siGENOME SMARTpool from Dharmacon, Thermo-Scientific, Waltham, USA and ThermoFisher Scientific), were transfected using a Monocyte Amaxa Nucleofection kit (Lonza, Basel, Switzerland) following manufacturer instructions. Monocytes were left untreated overnight and then differentiated to macrophages or dendritic cells as described above.

For activated CD4+ T cells, 9 pmol of the corresponding siRNA with 0.2 μl of siGuard RNase inhibitor (Genlantis) per 1 million of CD4+ T cells were transfected using Amaxa Human T Cell Nucleofector Kit (Lonza, Basel, Switzerland) following manufacturer’s recommendations. CD4+ T cells were left untreated for 20 hours and harvested afterwards for HIV-1 infection or processed for mRNA or protein expression.

### Drugs

3-Azido-3-deoxythymidine (zidovudine, AZT) was purchased from Sigma-Aldrich (Madrid, Spain). Raltegravir (RAL) was obtained from the NIH AIDS Research and Reference Reagent Program. MRT67307, a pharmacological inhibitor of IKKϵ and TBK1, was purchased from Selleckchem. When appropriate, differentiated macrophages were incubated with 100 ng/ml of lipopolisaccaride (LPS, Sigma-Aldrich) or 10 µg/ml of Poly I:C (Sigma-Aldrich), during4 hours at 37 °C.

### Quantitative RT-polymerase chain reaction (qRT-PCR)

For relative mRNA quantification, RNA was extracted using the NucleoSpin RNA II kit (Magerey-Nagel), as recommended by the manufacturer, including the DNase I treatment step. Reverse transcriptase was performed using the PrimeScript™ RT-PCR Kit (Takara). mRNA relative levels of all genes were measured by two-step quantitative RT-PCR and normalized to GAPDH mRNA expression using the DDCt method. Primers and DNA probes were purchased from Life Technologies (TaqMan gene expression assays).

Cytokine expression was evaluated by using the commercial TaqMan Human Cytokine Network array (4414255, Life Technologies), which included primers and probes for 28 different cytokine genes. mRNA relative levels of all cytokine genes were measured by two-step quantitative RT-PCR and normalized to GAPDH mRNA expression using the DDCt method.

### Viruses and virus infections

Envelope-deficient HIV-1 NL4-3 clone encoding IRES-GFP (NL4-3-GFP) was pseudotyped with VSV-G by cotransfection of HEK293T cells using polyethylenimine (Polysciences) as previously described^19,25^. Three days after transfection, supernatants were harvested, filtered and stored at −80 °C. Viral stocks were concentrated using Lenti-X concentrator (Clontech). Viruses were titrated by infection of TZM cells followed by GFP quantification by flow cytometry. R5 HIV-1 strain BaL was grown in stimulated PBMC and specifically titrated for its use in assays in MDM.

M-CSF MDM, GM-CSF MDM or DC were infected with VSV-pseudotyped HIV-1 NL4-3-GFP as described before^43^. Viral replication was measured in all cases two days later by flow cytometry (LSRII, BD Biosciences). Measurement of cell cytotoxicity was performed by flow cytometry, i.e., cells were gated as living or dead, according to flow cytometry FSC and SSC parameters. M-CSF MDM were infected with the R5 HIV-1 strain BaL. Every 3 days, l00 μl of culture supernatant were replaced by 100 μl of M-CSF supplemented, fresh complete medium with or without the corresponding drug. HIV production was analyzed seven days after infection by ELISA HIV-p24 antigen detection in culture supernatants (BioRad, Barcelona, Spain). In all cases, antiviral drugs were added simultaneously with the virus.

### Quantification proviral DNA and integrated provirus

MDM were infected with HIV-1 BaL and infections were stopped after 16 h to measure proviral DNA or at 20 h to measure viral integration as described before^19,25^. Briefly, DNA was extracted using a DNA extraction kit (Qiagen) and proviral DNA quantifications were performed using Gag amplification using the following primers and probe: forward 5′-CAAGCAGCCATGCAAATGTT-3′, reverse 5′-TGCACTGGATGCAATCTATCC-3′, and probe FAM 5′-AAAGAGACCATCAATGAGGAAGCTGCAGA-3′ TAMRA.

For integrated DNA, an Alu-Gag (HIV group-specific antigen) pre-amplification was performed after recommendation of the manufacturer by using the following primers: forward 5′-GCCTCCCAAAGTGCTGGGATTACAG-3′, and reverse 5′AGGGTTCCTTTGGTCCTTGT-3. Afterwards, samples were then followed by a Gag amplification by using the following primers and probe: forward 5′-CAAGCAGCCATGCAAATGTT-3′, reverse 5′-TGCACTGGATGCAATCTATCC-3′, and probe FAM 5′-AAAGAGACCATCAATGAGGAAGCTGCAGA-3′ TAMRA. Ct values for proviral DNA and integrated viral DNA were normalized using RNaseP as housekeeping gene by the ΔΔCt method. Infections were normalized to an untreated control. To ensure that measured proviral DNA was the product of infection and not result from DNA contamination of the viral stocks samples treated with RT inhibitor AZT (1 μM) were run in parallel. Raltegravir (2 μM) was used to control for post-RT steps.

HIV-1 RNA transcription was quantified by measuring multiple spliced viral RNA 40 hours post infection as described before^44^. RNA was extracted using the NucleoSpin RNA XS kit (Magerey-Nagel) including the DNase I treatment step and reverse transcription performed using the PrimeScript™ RT-PCR Kit (Takara). A specific set of primers and probe to amplify spliced *tat/rev/nef* mRNA were used (forward 5′-GGATCTGTCTCTGTCTCTCTCTCCACC-3′, reverse 5′-ACAGTCAGACTCATCAAGTTTCTCTATCAAAGCA-3′ and the dual-labeled fluorescent probe FAM 5′-TTCCTTCGGGCCTGTCGGGTCCC-3′ TAMRA) and mRNA relative levels were measured by two-step quantitative RT-PCR, normalized to *GAPDH* mRNA expression using the DDCt method^45^.

### Western blot

Cells were rinsed in ice-cold phosphate-buffered saline (PBS) and extracts prepared in lysis buffer (50 mM Tris HCl pH 7.5, 1 mM EDTA, 1 mM EGTA, 1 mM Na3VO4, 10 mM Na β-glycerophosphate, 50 mM NaF, 5 mM Na Pyrophosphate, 270 mM sucrose and 1% Triton X-100) supplemented with protease inhibitor (Roche) and 1 mM phenylmethylsulfonyl fluoride. Lysates were subjected to SDS-PAGE and transferred to a PVDF membrane (ImmunolonP, Thermo). The following antibodies were used for immunoblotting: anti-rabbit and anti-mouse horseradish peroxidase-conjugated secondary antibodies (1:5000; Pierce); anti-human Hsp90 (1:1000; 610418, BD Biosciences), anti-HIV-Gag (ab9071) and anti-GAPDH (ab9485) 1:2500 from abcam and anti-ADAR1 (14175), anti-MDA5 (5321), anti-phosphoSTAT1 (9167), anti-RIG-I (3743 l), anti-IRF3 (11904), anti-IRF7 (4920) and anti-PKR (12297) all 1:1000 from Cell Signaling. Western blot bands were quantified using Image Studio Software (LI-Cor biosciences).

### Quantification of CXCL10 in the culture supernatant

CXCL10 was quantified in macrophage culture supernatant using human IP-10 ELISA Kit (ab173194) at day 4-post differentiation and/or day 7-post infection following manufacturer instructions.

### Determination of ADAR1 A-to-I editing

ADAR1 deaminase function was determined by sequencing *NEIL1* or 5′ UTR of HIV-1 RNA regions previously described to be modified by ADAR1^7,46^. Total RNA was extracted and reversed transcribed to obtain cDNA as described above. *NEIL1* was amplified first using primers N1-TCOF 5′-TCCAGACCTGCTGGAGCTAT-3′, and N1-TCOR 5′-GGCCTTGGATTTCTTTTTG-3′, followed by a nested PCR with Forward N1-TCIF 5′-CCCAAGGAAGTGGTCCAGTTGG-3′, and Reverse 1-TCIR 5′-CTGGAACCAGATGGTACGGCC-3′ primers, as described in^46^. 5′ UTR of HIV-1 RNA was amplified with the following primers: forward 5′-GGGTCTCTCTGGTTAGA-3′, and reverse 5′-GGGTTCCCTAGTTAG-3′, as previously described^24^. PCR products were run on a 2% agarose gel to confirm band size of approximately 150 bp for *NEIL1* and 180 bp for 5′ UTR of HIV-1 before analyzing them by direct sequencing.

The extent of editing at each site was determined by using the electropherograms from sequencing reactions to estimate the relative amounts of A and G. Mean A to G ratio of at least three independent experiments was calculated.

### Antiviral activity of IFN-β antibody

Transfected macrophages were treated with a dose-dependent amounts of anti-human IFN-β antibody (LEAF Purified anti-human IFN-b, 514004, BioLegend) or the isotype control (LEAF™ Purified Mouse IgG1, κ Isotype Ctrl Antibody, 400123, BioLegend). Treated macrophages were infected with VSV-pseudotyped NL4-3-GFP and viral replication was measured two days later by flow cytometry (LSRII, BD Biosciences).

### Evaluation of antiviral activity of culture supernatants

Supernatants from transfected macrophages were collected 4 days post-transfection and stored at −30 °C. Non-transfected monocytes from a distinct donor were differentiated to macrophages in 96 well plates for 4 days. Macrophages were incubated with different amounts of culture supernatants (150 µl and dilutions 5-fold), followed by infection with VSV-pseudotyped NL4-3-GFP and viral replication was measured two days later by flow cytometry (LSRII, BD Biosciences).

### Statistical analysis

Experimental data are presented as mean ± SD. Paired Student’s t test was used for comparison between two groups, using the GraphPad Prism software. p-values lower than 0.05 were considered significant.

## Electronic supplementary material

Supplementary information

## References

[1] George, C. X., John, L. & Samuel, C. E. An RNA editor, adenosine deaminase acting on double-stranded RNA (ADAR1). *Journal of interferon & cytokine research: the official journal of the International Society for Interferon and Cytokine Research* 3**4**, 437–446, 10.1089/jir.2014.0001 (2014). 10.1089/jir.2014.0001 PMC4046350 24905200

[2] Nishikura K (2016). A-to-I editing of coding and non-coding RNAs by ADARs. Nature reviews. Molecular cell biology.

[3] Song, C., Sakurai, M., Shiromoto, Y. & Nishikura, K. Functions of the RNA Editing Enzyme ADAR1 and Their Relevance to Human Diseases. *Genes* **7**, 10.3390/genes7120129 (2016). 10.3390/genes7120129 PMC5192505 27999332

[4] Wang, Q., Li, X., Qi, R. & Billiar, T. RNA Editing, ADAR1, and the Innate Immune Response. *Genes* **8**, 10.3390/genes8010041 (2017). 10.3390/genes8010041 PMC5295035 28106799

[5] Liddicoat BJ (2015). RNA editing by ADAR1 prevents MDA5 sensing of endogenous dsRNA as nonself. Science.

[6] Samuel CE (2011). Adenosine deaminases acting on RNA (ADARs) are both antiviral and proviral. Virology.

[7] Doria M, Neri F, Gallo A, Farace MG, Michienzi A (2009). Editing of HIV-1 RNA by the double-stranded RNA deaminase ADAR1 stimulates viral infection. Nucleic acids research.

[8] Orecchini E (2017). ADAR1 restricts LINE-1 retrotransposition. Nucleic acids research.

[9] Wong SK, Lazinski DW (2002). Replicating hepatitis delta virus RNA is edited in the nucleus by the small form of ADAR1. Proceedings of the National Academy of Sciences of the United States of America.

[10] Clerzius G (2009). ADAR1 interacts with PKR during human immunodeficiency virus infection of lymphocytes and contributes to viral replication. Journal of virology.

[11] Clerzius G, Gelinas JF, Gatignol A (2011). Multiple levels of PKR inhibition during HIV-1 replication. Reviews in medical virology.

[12] Pfaller CK, Li Z, George CX, Samuel CE (2011). Protein kinase PKR and RNA adenosine deaminase ADAR1: new roles for old players as modulators of the interferon response. Current opinion in immunology.

[13] Akira S, Uematsu S, Takeuchi O (2006). Pathogen recognition and innate immunity. Cell.

[14] Brubaker SW, Bonham KS, Zanoni I, Kagan JC (2015). Innate immune pattern recognition: a cell biological perspective. Annual review of immunology.

[15] Crow YJ, Manel N (2015). Aicardi-Goutieres syndrome and the type I interferonopathies. Nature reviews. Immunology.

[16] Rice GI (2013). Assessment of interferon-related biomarkers in Aicardi-Goutieres syndrome associated with mutations in TREX1, RNASEH2A, RNASEH2B, RNASEH2C, SAMHD1, and ADAR: a case-control study. The Lancet. Neurology.

[17] Hartmann G (2017). Nucleic Acid Immunity. Advances in immunology.

[18] Tamura T, Yanai H, Savitsky D, Taniguchi T (2008). The IRF family transcription factors in immunity and oncogenesis. Annual review of immunology.

[19] Badia R (2016). The G1/S Specific Cyclin D2 Is a Regulator of HIV-1 Restriction in Non-proliferating Cells. PLoS pathogens.

[20] Ballana E (2009). Cell adhesion through alphaV-containing integrins is required for efficient HIV-1 infection in macrophages. Blood.

[21] Briggs SD (2001). HIV-1 Nef promotes survival of myeloid cells by a Stat3-dependent pathway. The Journal of biological chemistry.

[22] Pauls E (2014). Cell cycle control and HIV-1 susceptibility are linked by CDK6-dependent CDK2 phosphorylation of SAMHD1 in myeloid and lymphoid cells. Journal of immunology.

[23] Pujantell M (2016). Long-term HIV-1 infection induces an antiviral state in primary macrophages. Antiviral research.

[24] Ruiz A (2014). Characterization of the influence of mediator complex in HIV-1 transcription. The Journal of biological chemistry.

[25] Ruiz A (2015). Cyclin D3-dependent control of the dNTP pool and HIV-1 replication in human macrophages. Cell cycle.

[26] Clark K (2011). Novel cross-talk within the IKK family controls innate immunity. The Biochemical journal.

[27] Erickson AK, Gale M (2008). Regulation of interferon production and innate antiviral immunity through translational control of IRF-7. Cell research.

[28] Schlee M, Hartmann G (2016). Discriminating self from non-self in nucleic acid sensing. Nature reviews. Immunology.

[29] Crow YJ (2015). Characterization of human disease phenotypes associated with mutations in TREX1, RNASEH2A, RNASEH2B, RNASEH2C, SAMHD1, ADAR, and IFIH1. American journal of medical genetics. Part A.

[30] Takanohashi A (2013). Elevation of proinflammatory cytokines in patients with Aicardi-Goutieres syndrome. Neurology.

[31] Hartner JC, Walkley CR, Lu J, Orkin SH (2009). ADAR1 is essential for the maintenance of hematopoiesis and suppression of interferon signaling. Nature immunology.

[32] Wang Q, Khillan J, Gadue P, Nishikura K (2000). Requirement of the RNA editing deaminase ADAR1 gene for embryonic erythropoiesis. Science.

[33] Kato H (2008). Length-dependent recognition of double-stranded ribonucleic acids by retinoic acid-inducible gene-I and melanoma differentiation-associated gene 5. The Journal of experimental medicine.

[34] Kato H (2006). Differential roles of MDA5 and RIG-I helicases in the recognition of RNA viruses. Nature.

[35] Takeuchi O, Akira S (2008). MDA5/RIG-I and virus recognition. Current opinion in immunology.

[36] Mannion NM (2014). The RNA-editing enzyme ADAR1 controls innate immune responses toRNA. . Cell reports.

[37] Cuadrado E (2015). ADAR1 Facilitates HIV-1 Replication in Primary CD4+ T Cells. PloS one.

[38] Cachat A (2014). ADAR1 enhances HTLV-1 and HTLV-2 replication through inhibition of PKR activity. Retrovirology.

[39] Iizasa H (2010). Editing of Epstein-Barr virus-encoded BART6 microRNAs controls their dicer targeting and consequently affects viral latency. The Journal of biological chemistry.

[40] Li Z, Wolff KC, Samuel CE (2010). RNA adenosine deaminase ADAR1 deficiency leads to increased activation of protein kinase PKR and reduced vesicular stomatitis virus growth following interferon treatment. Virology.

[41] Nie Y, Hammond GL, Yang JH (2007). Double-stranded RNA deaminase ADAR1 increases host susceptibility to virus infection. Journal of virology.

[42] Cuadrado E (2015). Phenotypic variation in Aicardi-Goutieres syndrome explained by cell-specific IFN-stimulated gene response and cytokine release. Journal of immunology.

[43] Badia R (2017). SAMHD1 is active in cycling cells permissive to HIV-1 infection. Antiviral Research.

[44] Ballana E (2010). Zinc ribbon domain-containing 1 (ZNRD1) is a host cellular factor influencing HIV-1 replication and disease progression. Clinical Infectious Diseases.

[45] Ciuffi A (2004). Entry and transcription as key determinants of differences in CD4 T-cell permissiveness to human immunodeficiency virus type 1 infection. Journal of virology.

[46] Yeo J, Goodman RA, Schirle NT, David SS, Beal PA (2010). RNA editing changes the lesion specificity for the DNA repair enzyme NEIL1. Proceedings of the National Academy of Sciences of the United States of America.

