# Supplementary material for: RNA editing by ADAR1 regulates innate and antiviral immune functions in primary macrophages
Source: Sci Rep. 2017 Oct 17;7:13339. doi: 10.1038/s41598-017-13580-0 (PMC5645456; doi:10.1038/s41598-017-13580-0)

Supplementary information

RNA editing by ADAR1 regulates innate and antiviral immune functions in primary macrophages

Maria Pujantell[1], Eva Riveira-Muñoz[1], Roger Badia[1], Marc Castellví[1], Edurne Garcia-Vidal[1], Guillem Sirera[2], Teresa Puig[1], Cristina Ramirez[1], Bonaventura Clotet[1], José A. Esté[1,*] and Ester Ballana[1,*]

Supplementary Figure Legends

Supplementary Figure 1. ADAR1 knockdown boosts type I IFN response and blocks HIV-1 replication in primary macrophages. (A) Effective downregulation of *ADAR1* by two different siRNA in primary macrophages. Relative mRNA expression of *ADAR1* was measured by quantitative PCR and normalized to GAPDH expression. Data represents mean ± SD of 3 different donors and is normalized to Mock-transfected macrophages. **(B)** Relative mRNA expression of *IFIH1*, *IFNB1* and *CXCL10* in ADAR1 knockdown macrophages by two different siRNA. mRNA expression was measured by quantitative PCR and normalized to GAPDH expression. Data represents mean ± SD of 3 different donors and is normalized to Mock-transfected macrophages. Protein expression in ADAR1 and IFIH1 knockdown macrophages. **(C)** HIV-1 replication in ADAR1 knockdown M-CSF macrophages, infected with a VSV-pseudotyped, GFP-expressing HIV-1. Data represent percentage replication relative to mock-transfected macrophages. Mean ± SD of 3 different donors performed in triplicate is shown. **(D)** Western blot showing ADAR1 downregulation by two independent siRNA and innate immune activation following ADAR1 knockdown. GAPDH was used as loading control. A representative donor is shown. The figure shows the cropped gels/blots obtained by each protein evaluation. Full-length blots of each tested protein are included in supplementary material. * $p<0.05$; ** $p<0.005$; *** $p<0.0005$.

Supplementary Figure 2. ADAR1 efficiently edits host *NEIL1* transcript, but does not edit HIV-1 *TAR* transcript. (A) Representative DNA sequencing chromatogram of RT-PCR products of 5' UTR HIV-1 mRNA transcript and the estimated percentage of editing efficiency at positions TAR520/521, TAR551/552 and TAR590. **(B-C)** Representative DNA

sequencing chromatograms of of *NEIL1* transcripts in M-CSF (B) and GM-CSF (C) MDMs of siNT and siADAR1, and relative ADAR1 editing efficiency at edited site. 5’ UTR HIV-1 could not be amplified in si*ADAR1* sample due to restricted infection. Edited Adenosines to Inosine are detected as G by direct sequencing. *p<0.05; **p<0.0005.

Supplementary Figure 3. PKR knockdown by siRNA does not affect HIV-1 replication or innate immune function (A) Relative PKR mRNA (left panel) and protein (right panel) expression is enhanced in ADAR1 knockdown macrophages. mRNA expression data represents mean ± SD of 3 different donors and is normalized to Mock-transfected macrophages. * p<0.05. Western blot shows data from a representative donor. **(B)** Effective downregulation of PKR by siRNA does not effect mRNA expression of innate immune receptors or effectors. mRNA expression of *PKR*, *ADAR1*, *IFNB1*, *IFIH1*, *DDX58*, *IRF3* and *IRF7* was measured by qPCR and expression is normalized to mock-transfected macrophages. Data represents mean ± SD of 3 different donors. **(C)** Western blot showing PKR downregulation and innate immune proteins in PKR knockdown macrophages. GAPDH was used as loading control. A representative donor is shown. **(D)** HIV-1 replication in PKR knockdown M-CSF macrophages, infected with a VSV-pseudotyped, GFP-expressing HIV-1. Data represent percentage replication relative to mock-transfected macrophages. Mean ± SD of 3 different donors performed in triplicate is shown. The figure shows the cropped gels/blots obtained by each protein evaluation. Full-length blots of each tested protein are included in supplementary material.

Supplementary Figure 4. ADAR1 knockdown macrophages maintain signatures of innate immune activation after long-term infection. (A) Relative mRNA expression of IFNβ in siRNA-treated macrophages 7 days post infection with HIV-1 BaL. *IFNB1* gene

expression was still significantly enhanced in ADAR1 knockdown macrophages. Data represents mean ± SD of 3 different donors and is normalized to Mock-transfected M-CSF macrophages. **(B)** CXCL10 mRNA (left panel) and protein expression in the supernatant (right panel) in siRNA-treated macrophages 7 days post infection with HIV-1 BaL. Relative mRNA expression of *CXCL10* was measured by quantitative PCR and normalized to GAPDH expression. CXCL10 protein in the culture supernatants was measured by ELISA. Data represents mean ± SD of 3 different donors. * p<0.0484; ** p<0.0098. **(C)** Protein expression in ADAR1 knockdown macrophages 7 days post infection with HIV-1 BaL. Western blot of ADAR1, phosphorylation of STAT1 (pSTAT1) and HIV-GAG in siRNA-treated infected macrophages. pSTAT1 is increased in ADAR1 knockdown macrophages compared to mock or non-targeting siRNA (NT). GAPDH was used as loading control. A representative donor is shown. The figure shows the cropped gels/blots obtained by each protein evaluation. Full-length blots of each tested protein are included in supplementary material.

Supplementary Figure 5. ADAR1-mediated regulation of innate immune activation and HIV-1 infection is specific of macrophages. (A) Relative mRNA expression of *IFIH1*, *IFNB1, CXCL10*, *IRF3* and *IRF7* in ADAR1 knockdown GM-CSF macrophages. mRNA expression was measured by quantitative PCR and normalized to GAPDH expression. Data represents mean ± SD of 3 different donors and is normalized to Mock-transfected macrophages. **(B)** Relative mRNA expression of *IFIH1*, *IFNB1* and *CXCL10* in ADAR1 knockdown dendritic cells. mRNA expression was measured by quantitative PCR and normalized to GAPDH expression. Data represents mean ± SD of 3 different donors and is normalized to Mock-transfected macrophages. * p<0.05; ** p<0.005.

Supplementary Figure 6. (A) ADAR1 knockdown macrophages induce overexpression of MDA5, RIG-I, pSTAT1 and IRF7. Quantification of Western blot bands showing the effect of ADAR1 knockdown in MDA5, RIG-I, pSTAT1, IRF7 and IRF3 expression relative to mock-transfected macrophages, corresponding to Fig 4B (left panel) and 4C (right panel). **(B) Blockade of TBK1 function restores protein expression phenotype observed in ADAR1 expressing macrophages.** Protein expression in ADAR knockdown macrophages treated with growing concentrations of the TBK1 inhibitor MRT67307 (5 µM, 1 µM, 0.2 µM and 0.04 µM). A representative donor is shown. ND, no drug. The figure shows the cropped gels/blots obtained by each protein evaluation. Full-length blots of each tested protein are included in supplementary material.

Supplementary Figure 7. ADAR1-mediated inhibition of HIV-1 replication is not affected by secreted IFNβ. Percentage HIV-1 infection in ADAR1 knockdown macrophages, untreated (black bars) or treated with different concentrations of anti-IFNβ1 (grey bars) or an isotype control (white bars). No change in HIV-1 susceptibility was observed in ADAR1, in mock-transfected macrophages or macrophages transfected with a non-targeting siRNA. Data represents mean ± SD of 3 different donors performed in triplicate.

Supplementary Figure 1

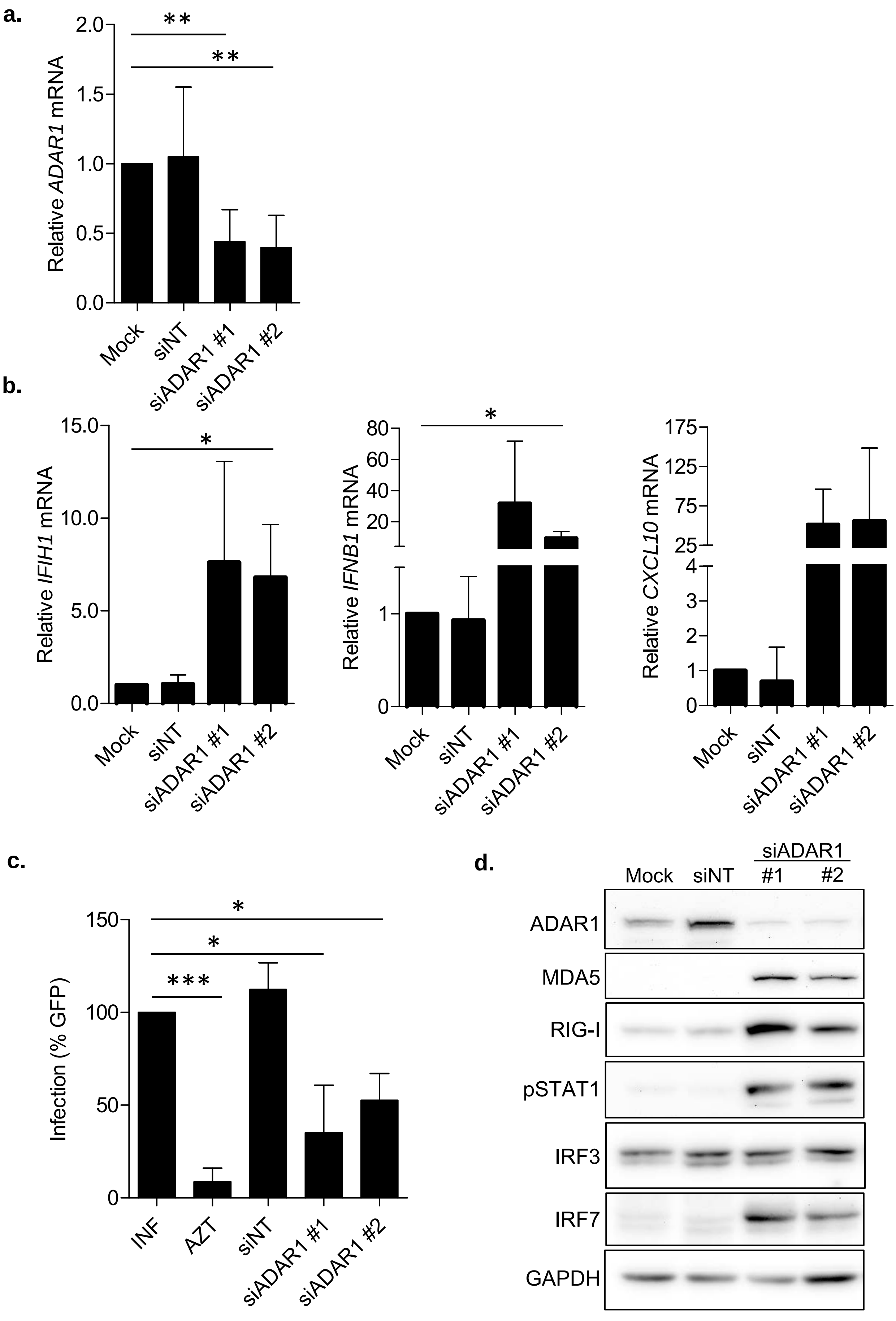

Supplementary Figure 2

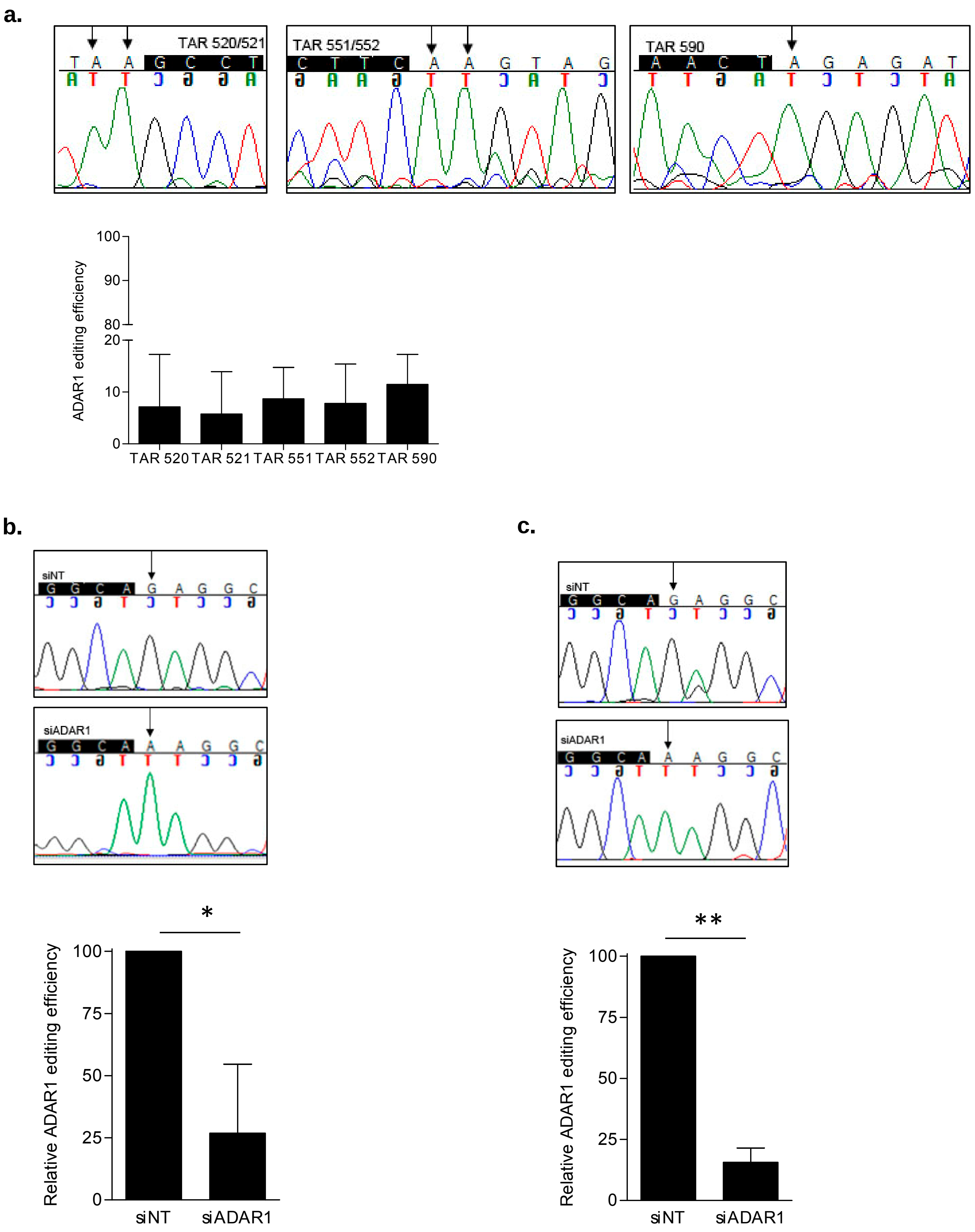

Supplementary Figure 3

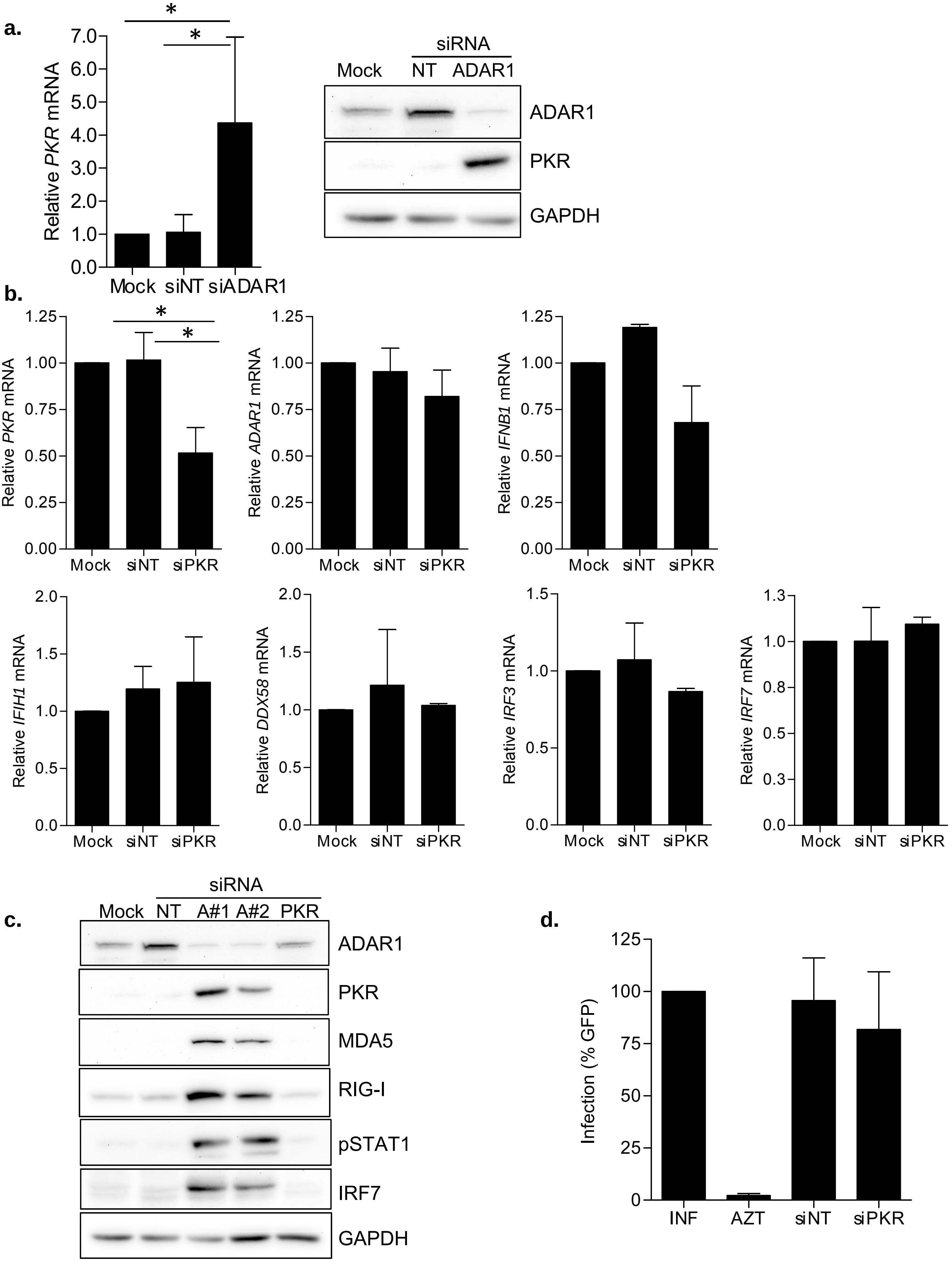

Supplementary Figure 4

a.

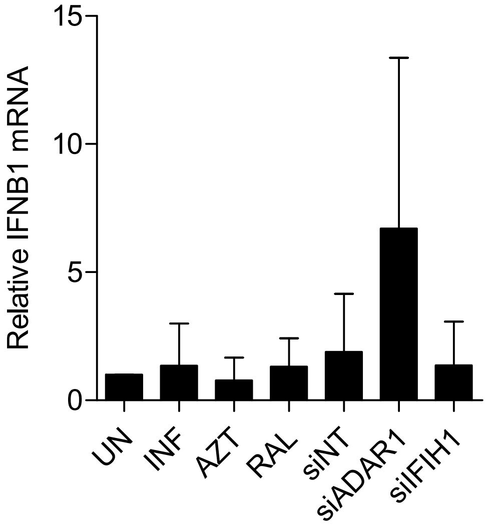

b.

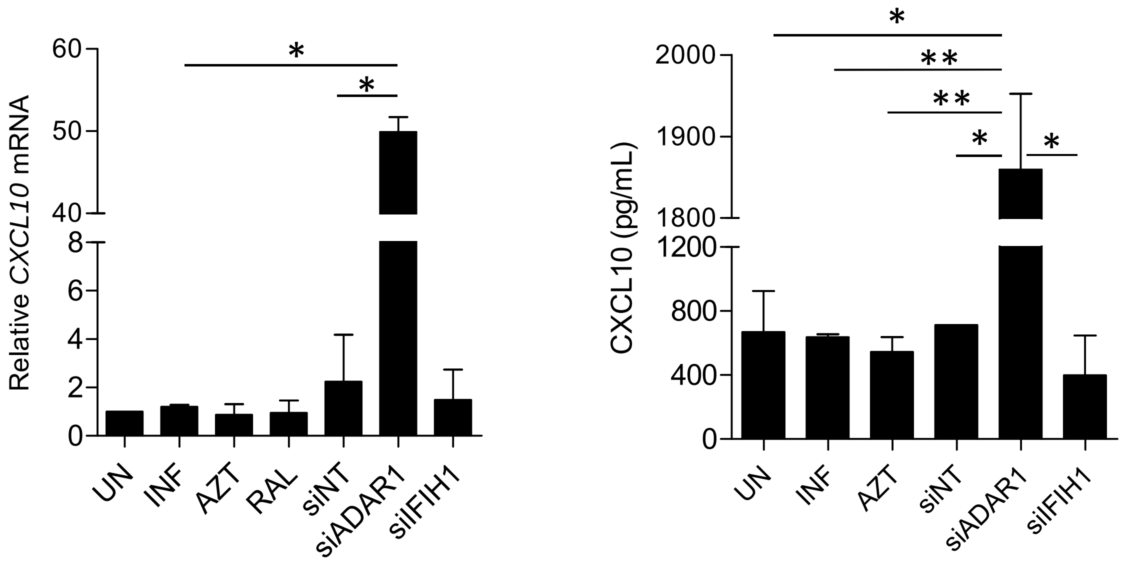

c.

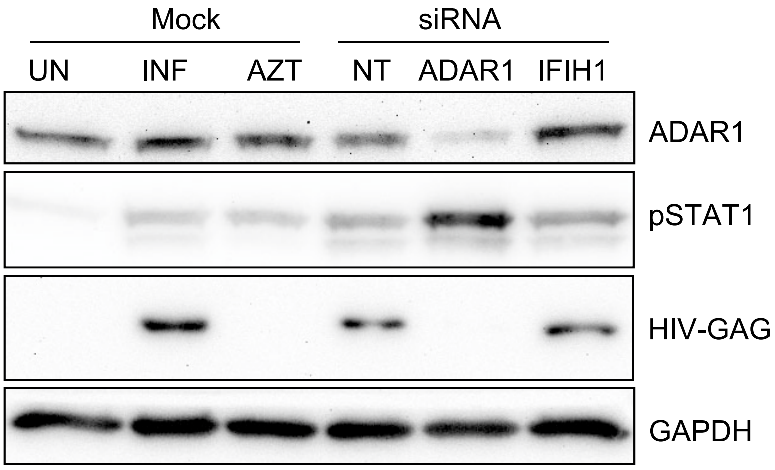

Supplementary Figure 5

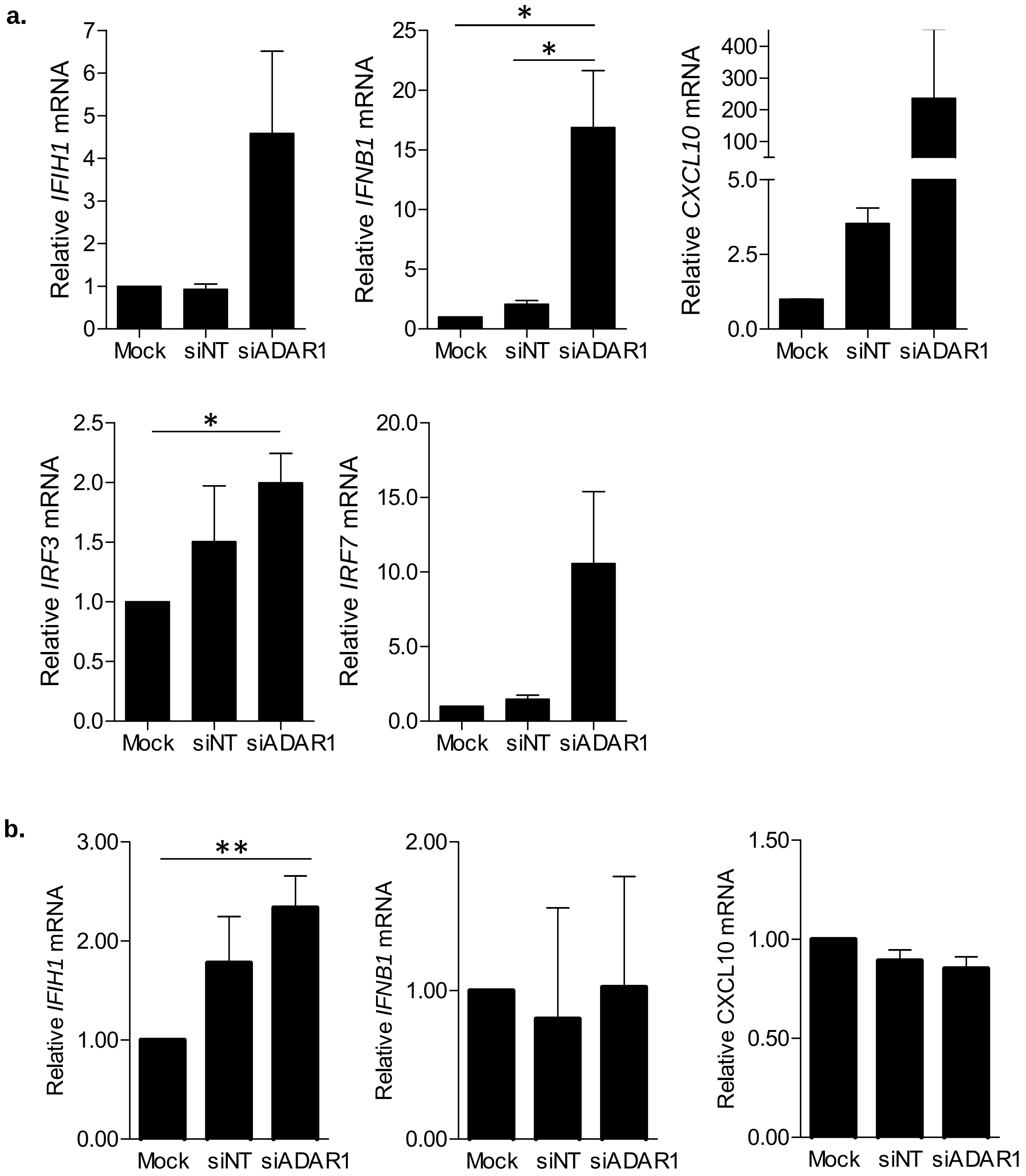

Supplementary Figure 6

a.

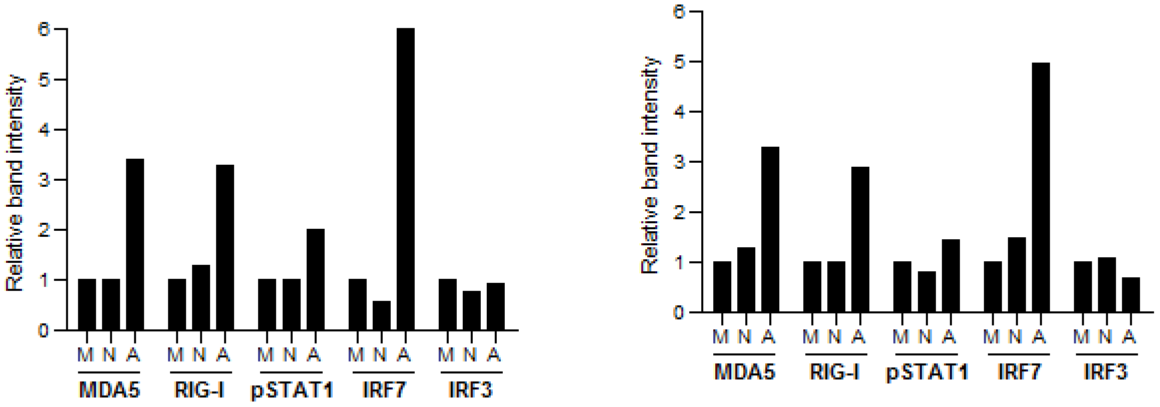

b.

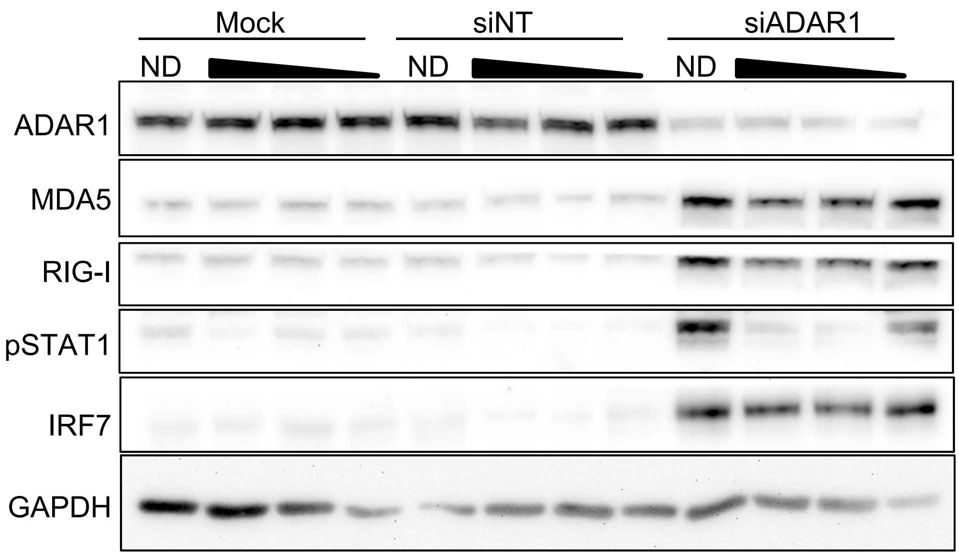

Supplementary Figure 7

a.

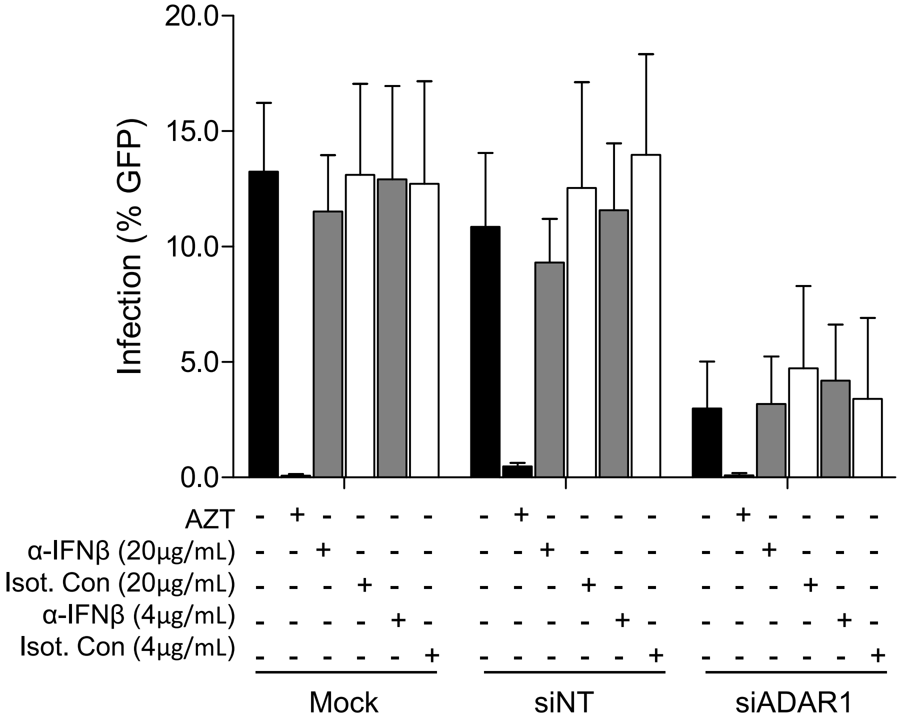

Supplementary Western Blot Images

Full-length Western blot to Fig 1B

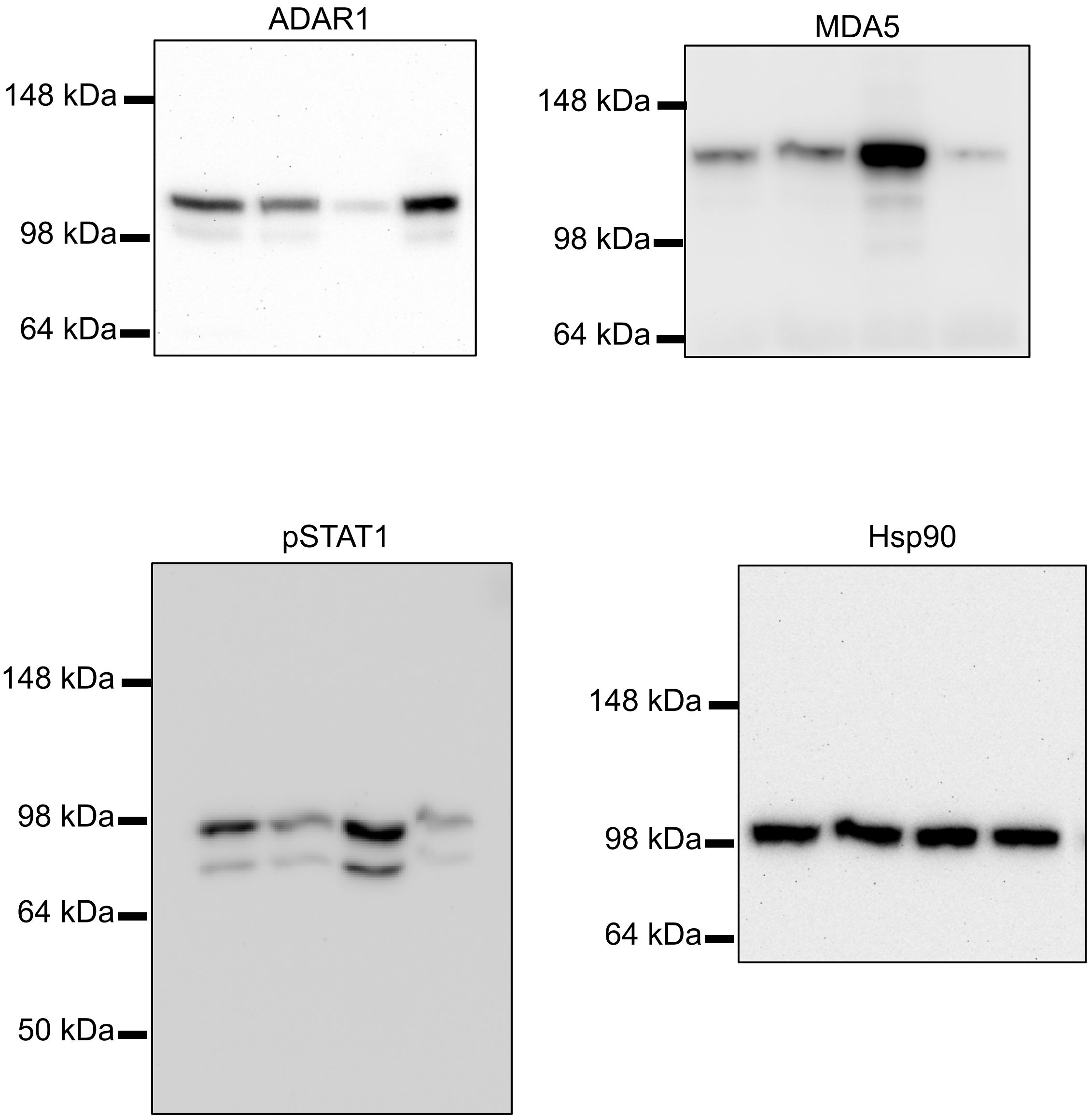

Full-length Western blot to Fig 3A

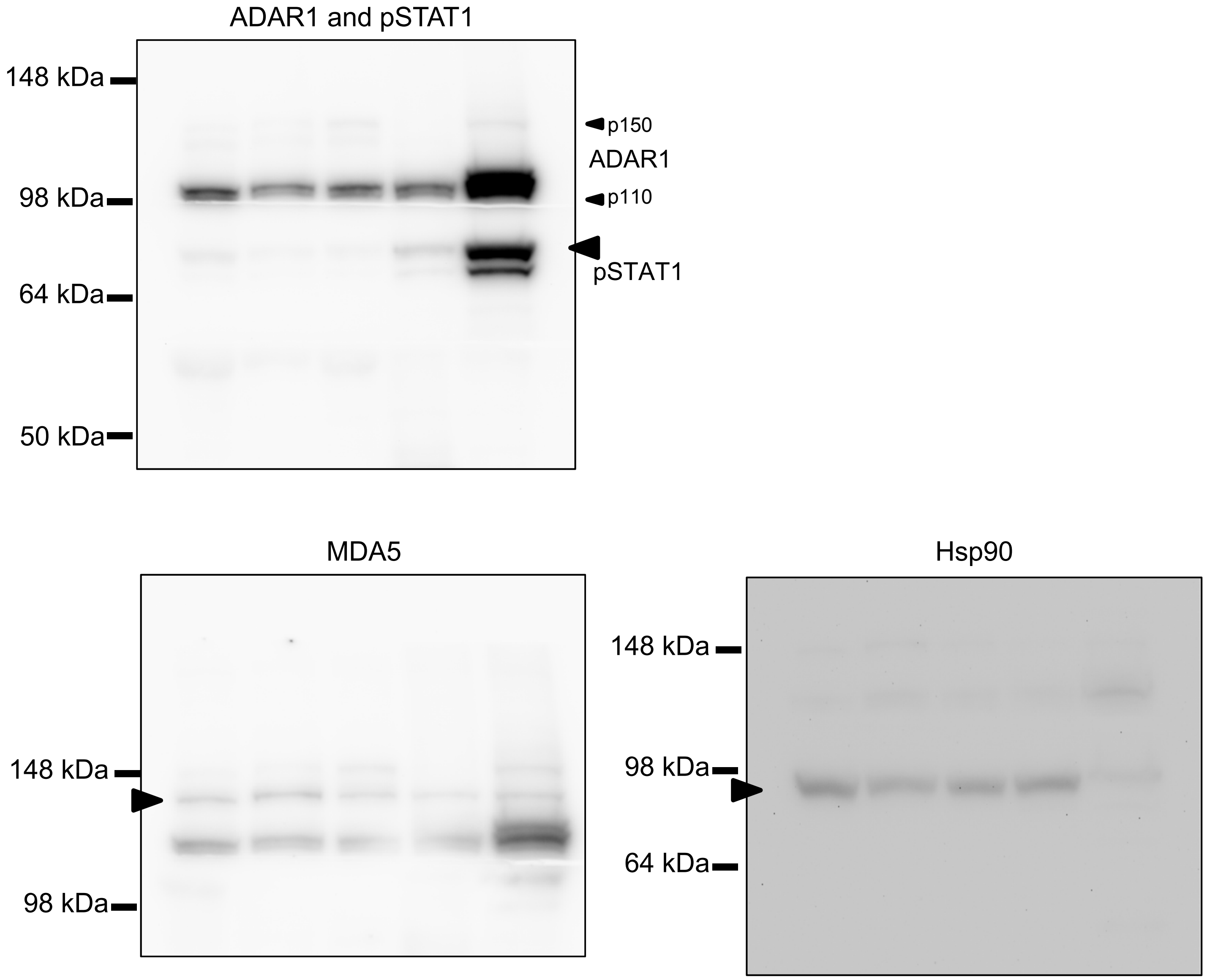

Full-length Western blot to Fig 3B

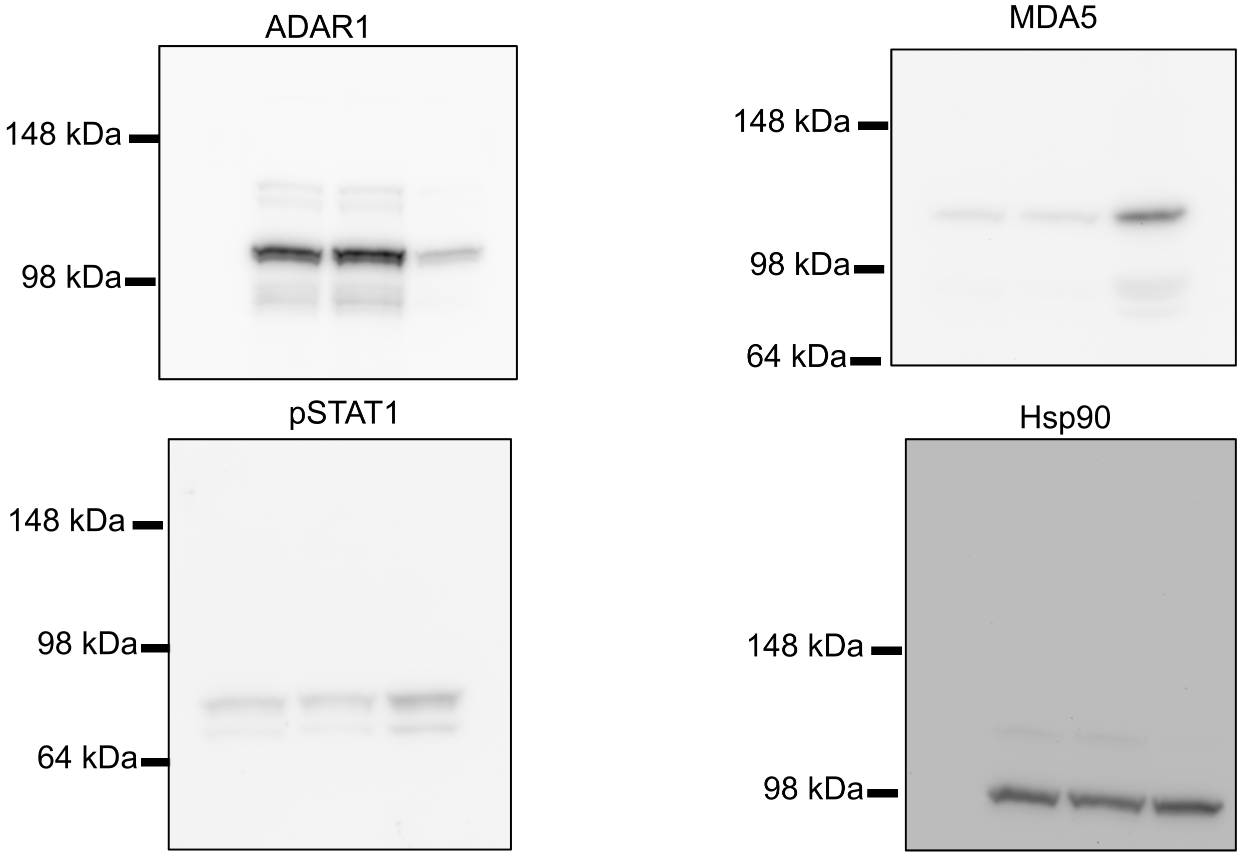

Full-length Western blot to Fig 3C

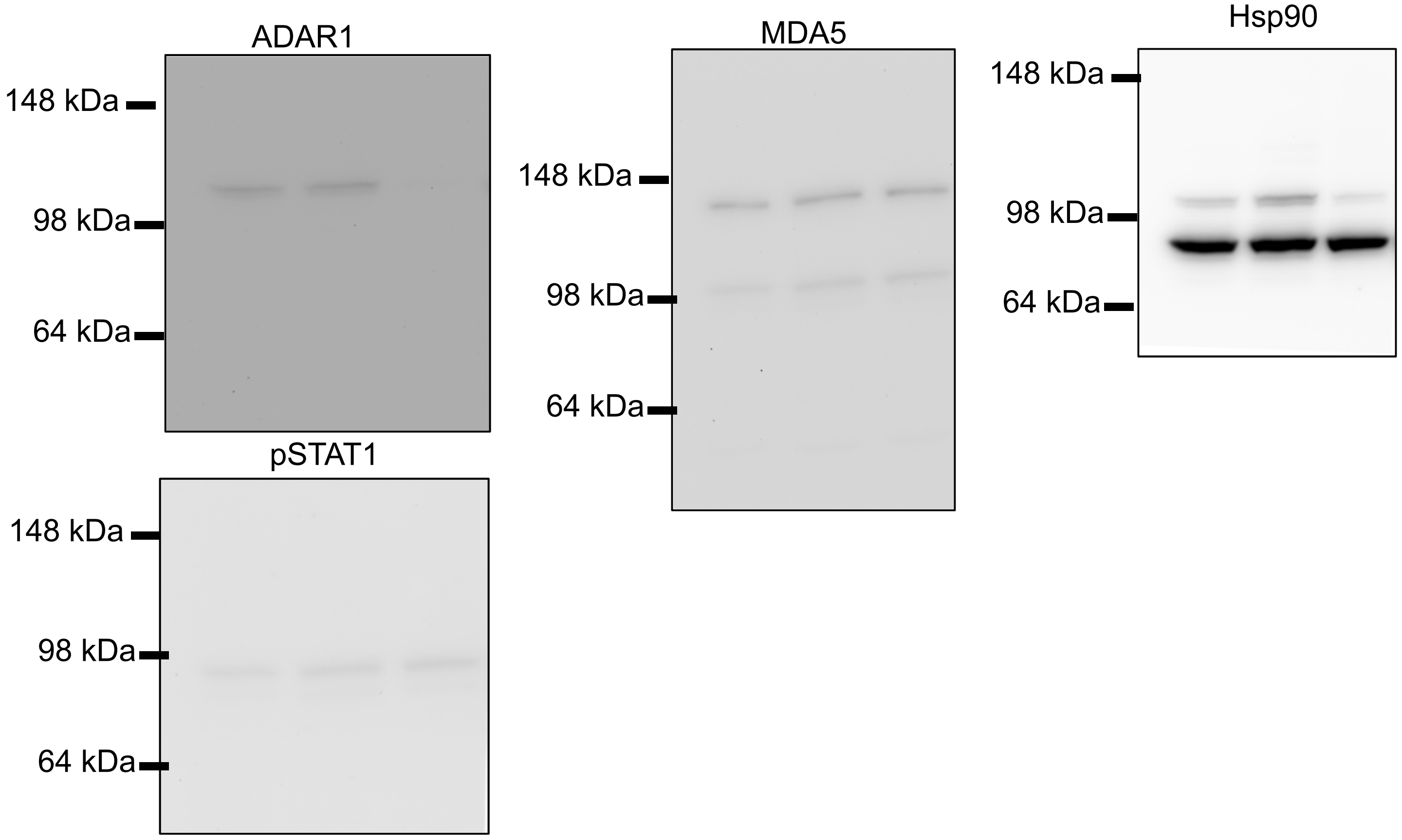

Full-length Western blot to Fig 3D

ADAR1

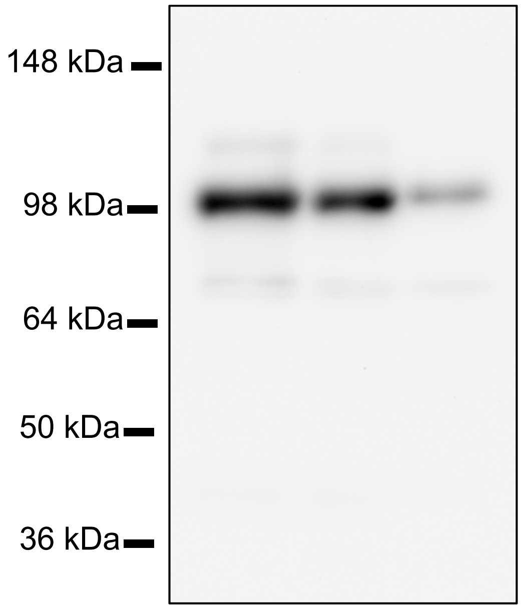

MDA5 and pSTAT1

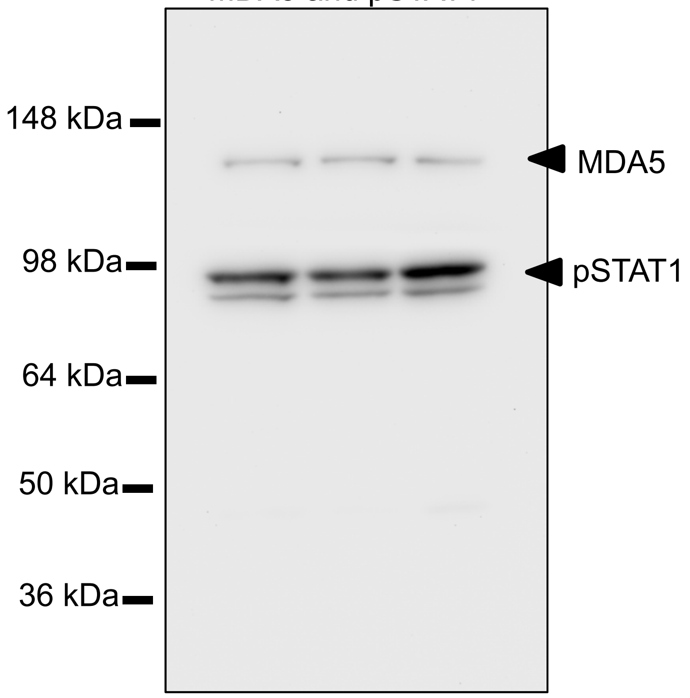

GAPDH

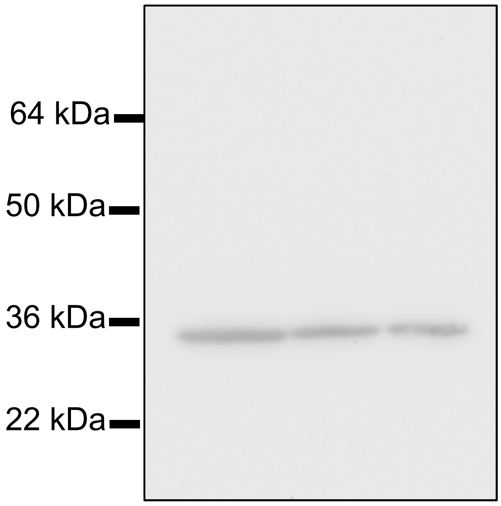

Full-length Western blot to Fig 4B

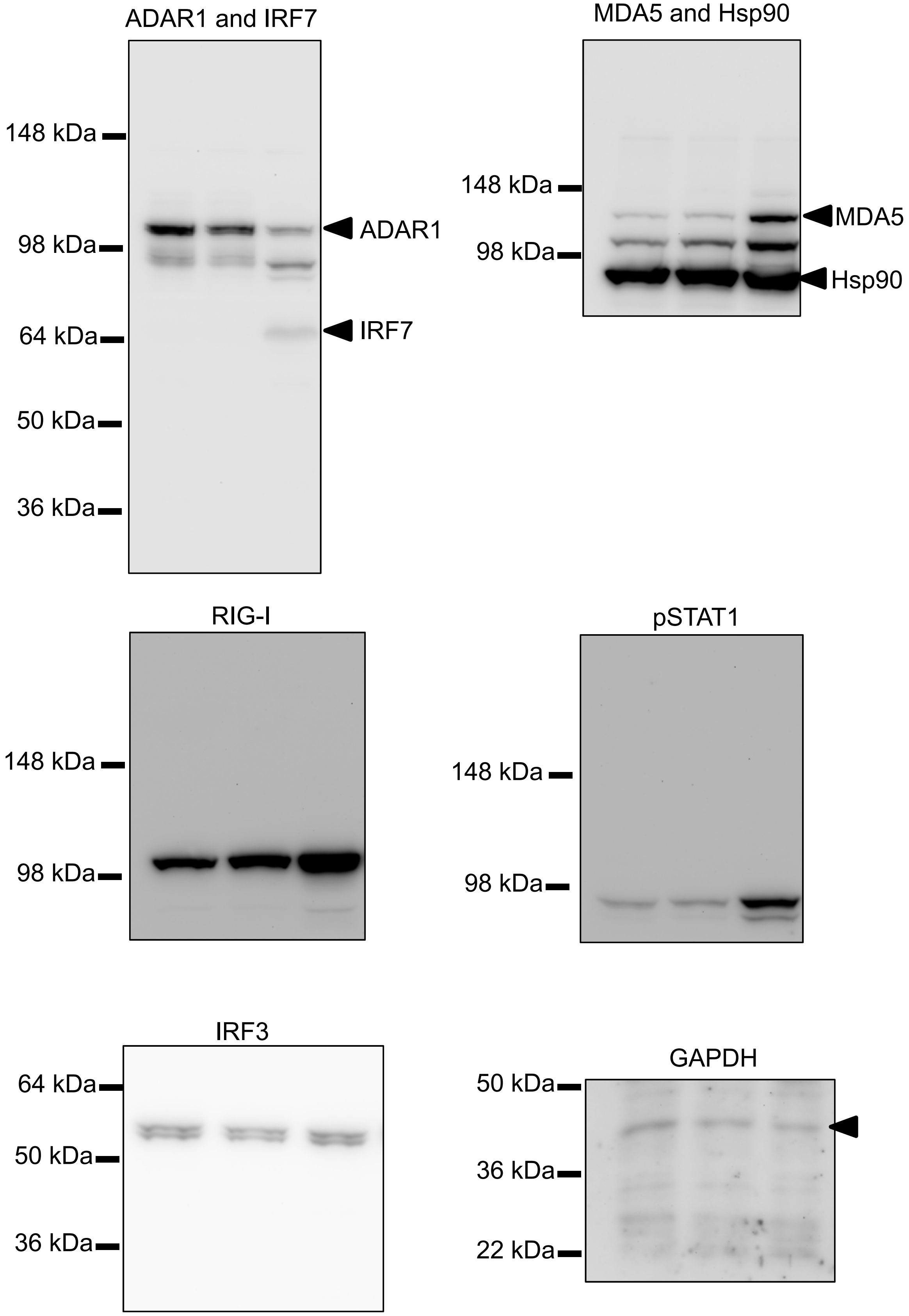

Full-length Western blot to Fig 4C

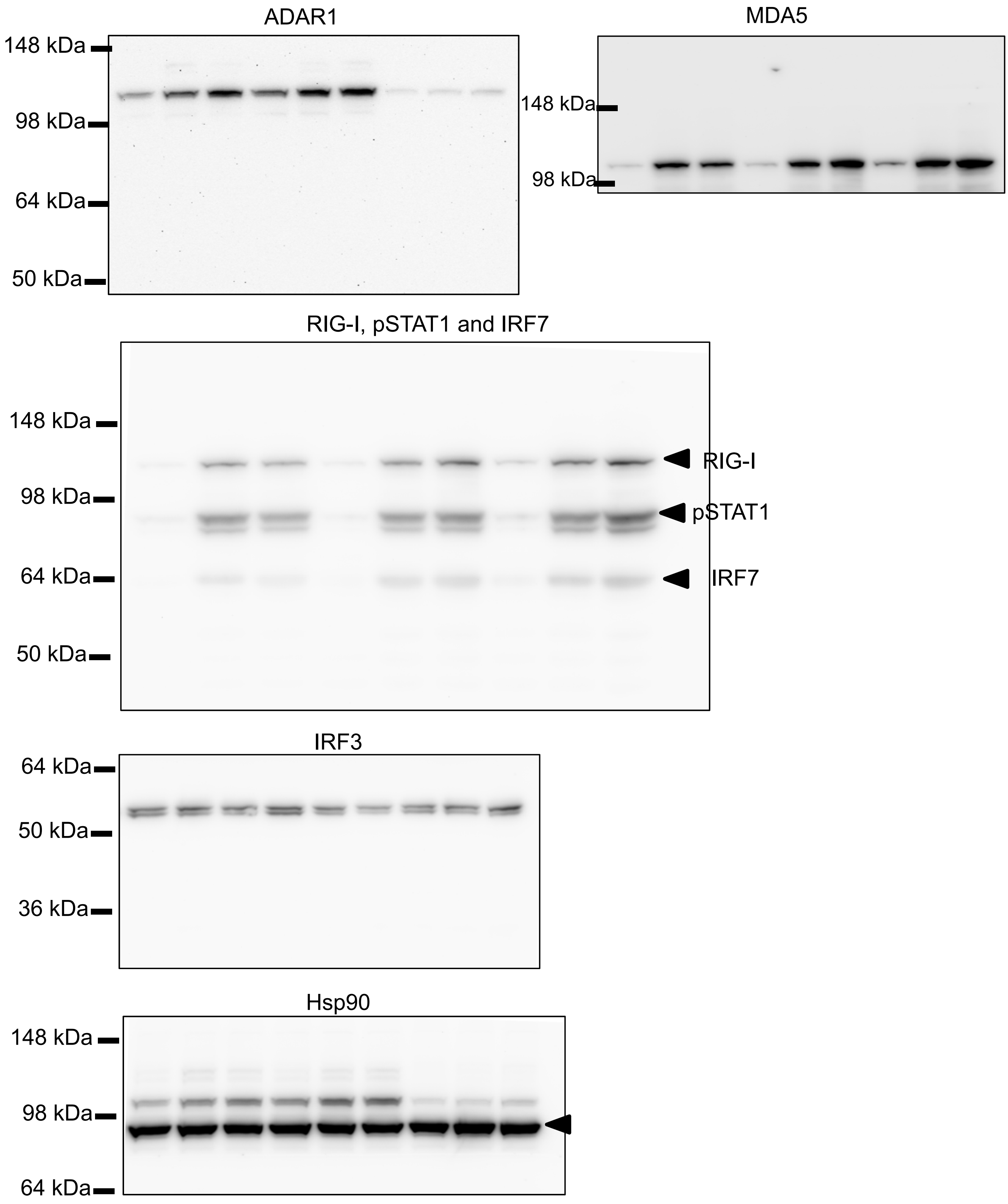

Full-length Western blot to Fig 4D

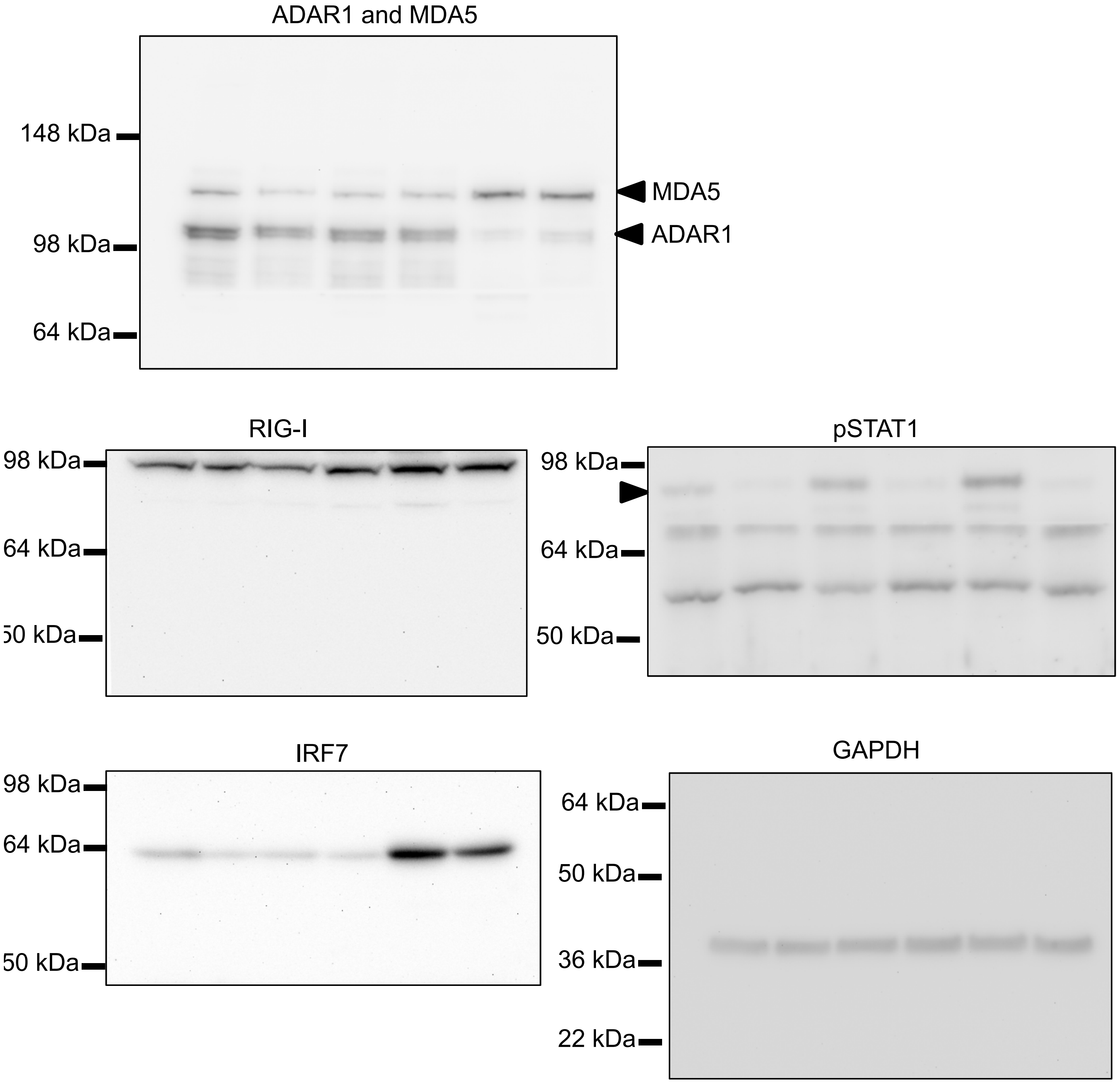

Full-length Western blot to Supplementary Fig 1D

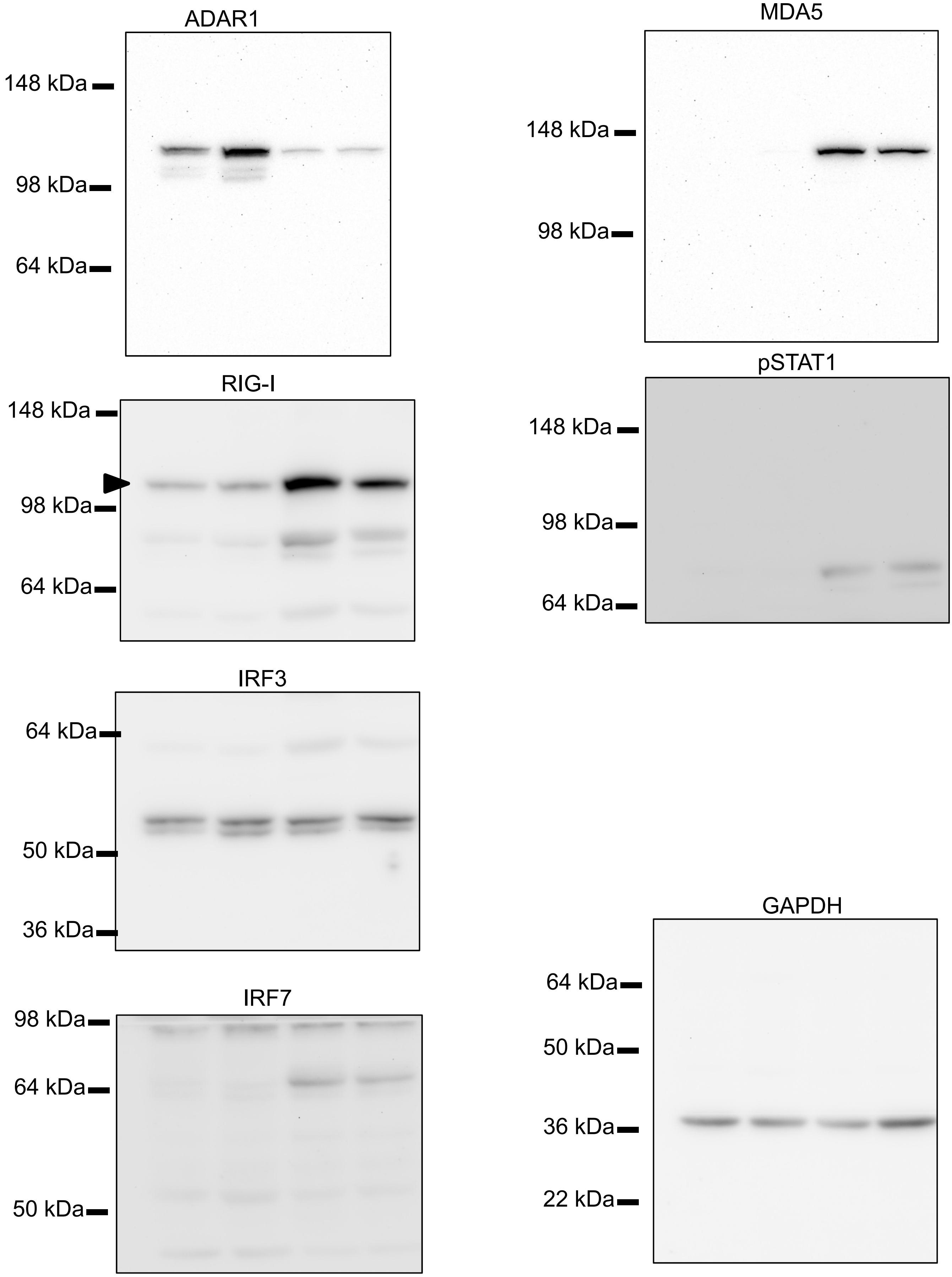

Full-length Western blot to Supplementary Fig 3A

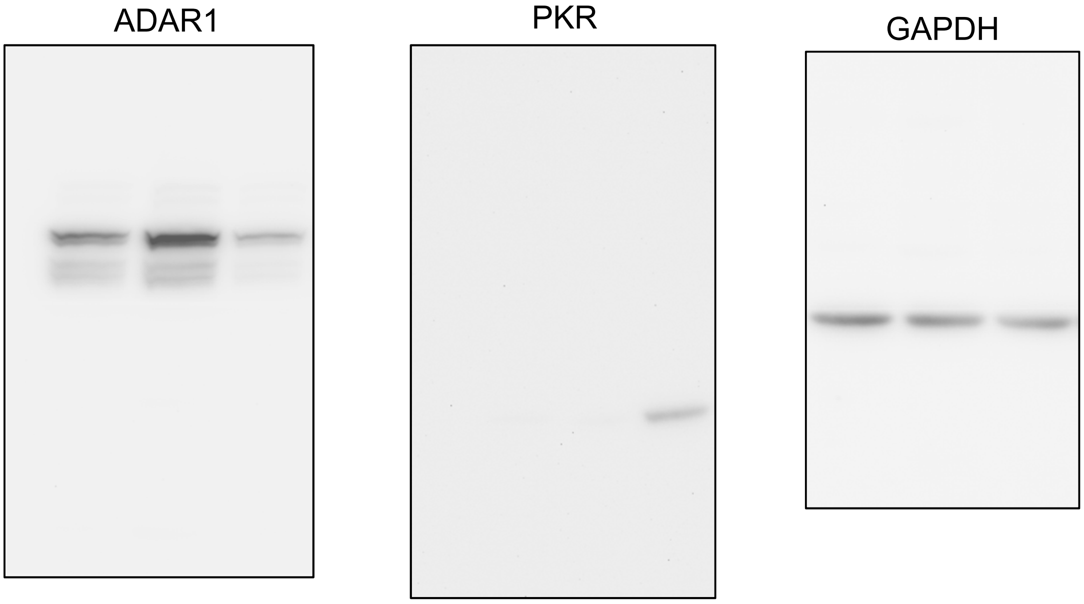

Full-length Western blot to Supplementary Fig 3C

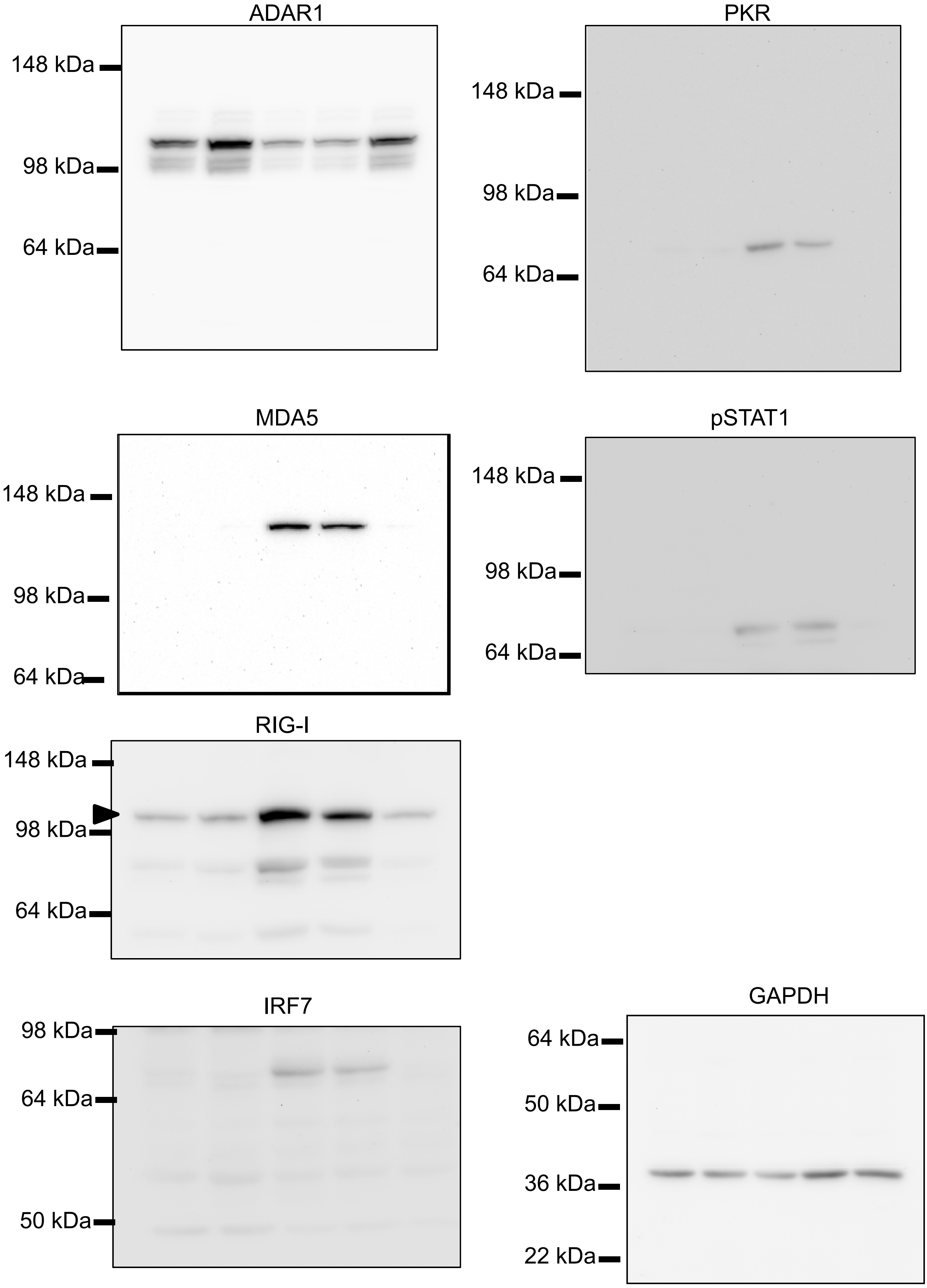

Full-length Western blot to Supplementary Fig 4C

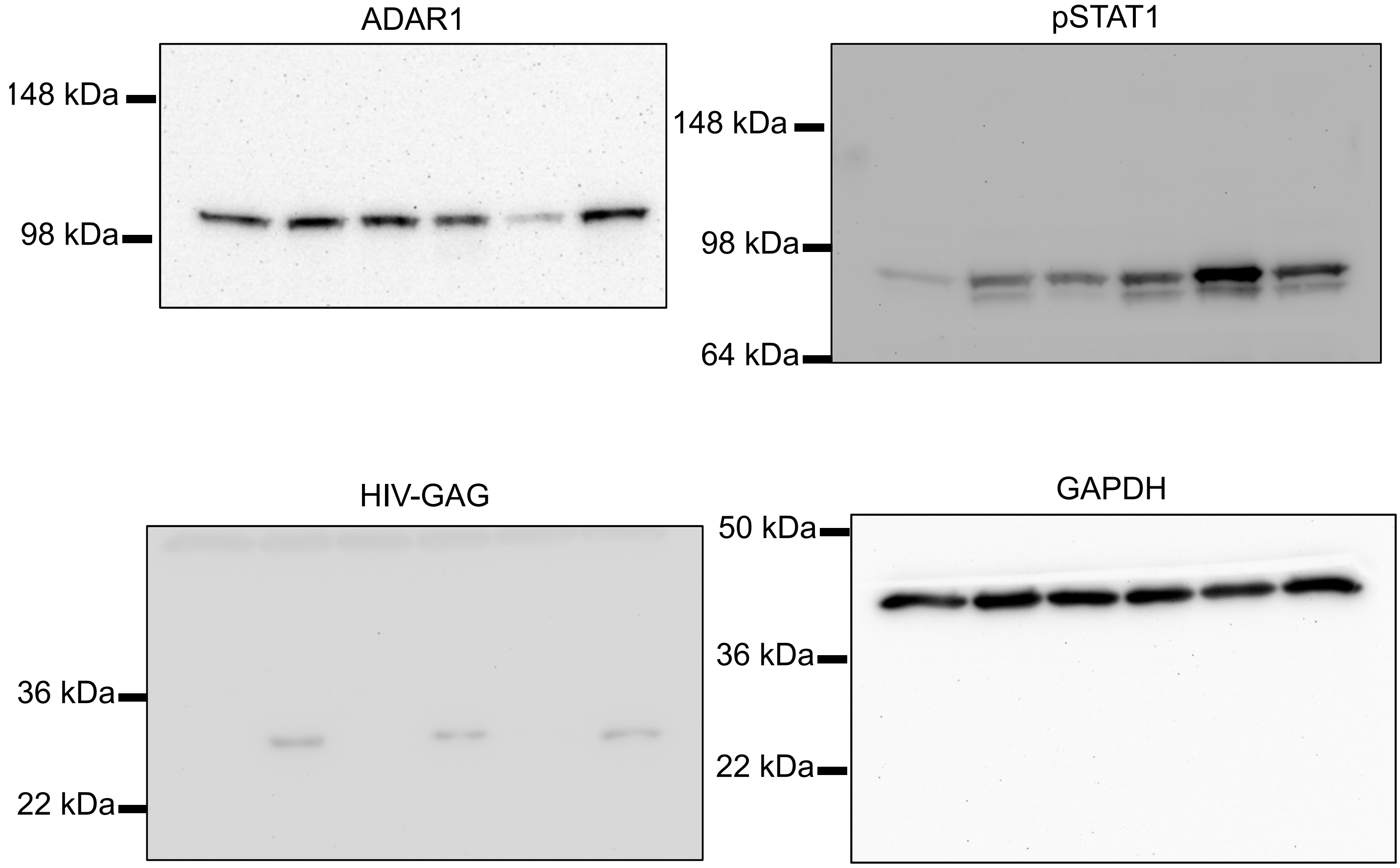

Full-length Western blot to Supplementary Fig 6

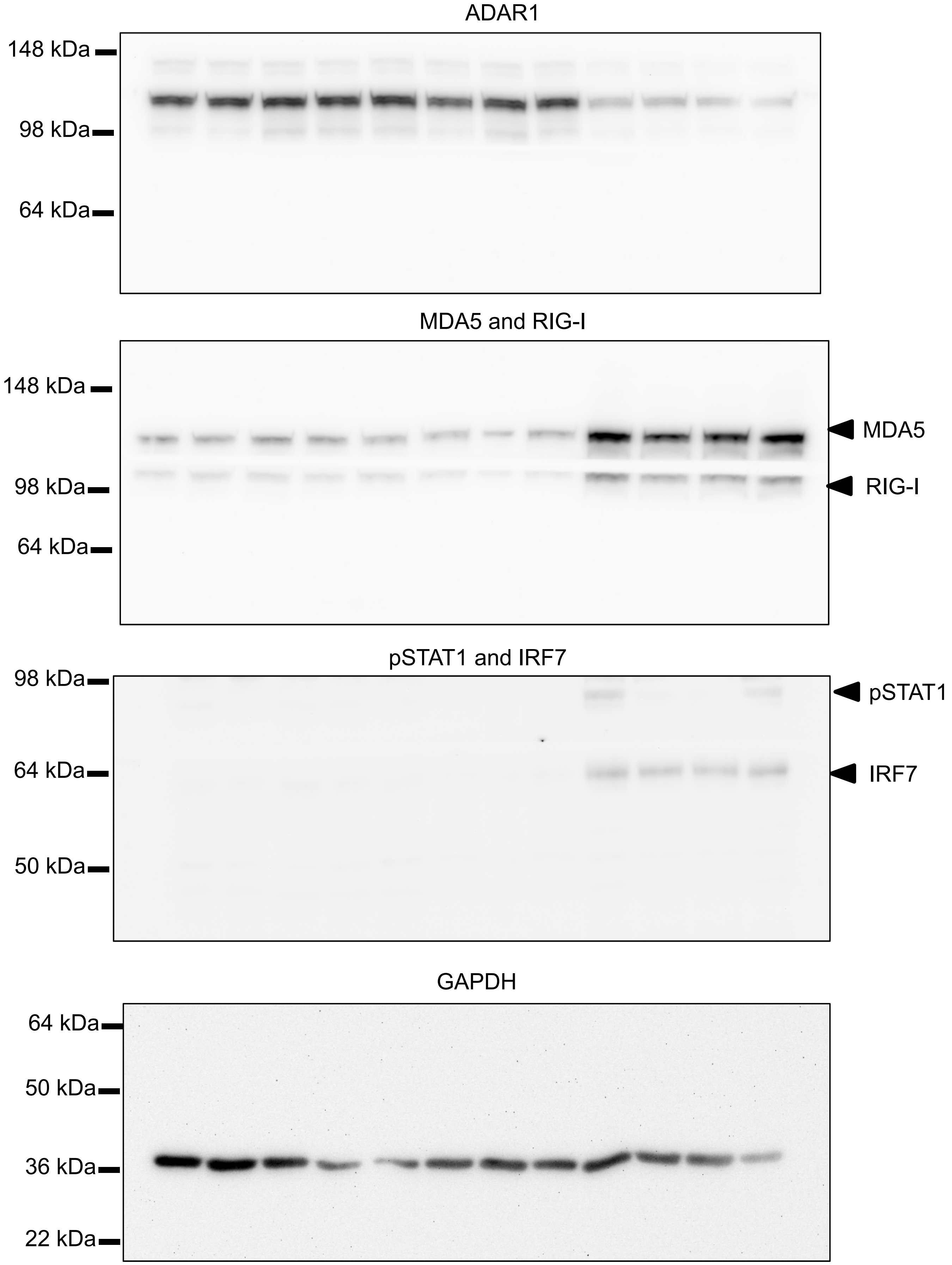

Supplement: Supplementary file 1 — Supplementary information [file 41598_2017_13580_MOESM1_ESM.pdf]
